# Evidence on physical activity and osteoporosis prevention for people aged 65+ years: a systematic review to inform the WHO guidelines on physical activity and sedentary behaviour

**DOI:** 10.1186/s12966-020-01040-4

**Published:** 2020-11-26

**Authors:** Marina B. Pinheiro, Juliana Oliveira, Adrian Bauman, Nicola Fairhall, Wing Kwok, Catherine Sherrington

**Affiliations:** 1 Institute for Musculoskeletal Health, The University of Sydney and Sydney Local Health District, Sydney, Australia; 2 School of Public Health, Faculty of Medicine and Health, The University of Sydney, Sydney, Australia; 3 Prevention Research Collaboration, Sydney School of Public Health, The University of Sydney, Sydney, Australia

**Keywords:** Physical activity, Osteoporosis, Bone mineral density, Older people, Review

## Abstract

**Background:**

Various physical activity interventions for prevention and treatment of osteoporosis have been designed and evaluated, but the effect of such interventions on the prevention of osteoporosis in older people is unclear. The aim of this review was to investigate the association between physical activity and osteoporosis prevention in people aged 65 years and above.

**Methods:**

A systematic review was conducted and searches for individual studies were conducted in PubMed (January 2010 to March 2020) and for systematic reviews were conducted in PubMed, Embase, CINAHL and SPORTDiscus (January 2008 to July 2020). Records were screened according to the following eligibility criteria: i) population: adults aged 65 years and older; ii) exposure: greater volume, duration, frequency, or intensity of physical activity; iii) comparison: no physical activity or lesser volume, duration, frequency, or intensity of physical activity; iv) outcome: osteoporosis related measures (e.g., bone mineral density). The methodological quality of included studies was assessed and meta-analysis summarised study effects. The GRADE approach was used to rate certainty of evidence.

**Results:**

We included a total of 59 studies, including 12 observational studies and 47 trials. Within the included trials, 40 compared physical activity with no intervention controls, 11 compared two physical activity programs, and six investigated different doses of physical activity. Included studies suggest that physical activity interventions probably improve bone health among older adults and thus prevent osteoporosis (standardised effect size 0.15, 95% CI 0.05 to 0.25, 20 trials, moderate-certainty evidence, main or most relevant outcome selected for each of the included studies). Physical activity interventions probably improve lumbar spine bone mineral density (standardised effect size 0.17, 95% CI 0.04 to 0.30, 11 trials, moderate-certainty evidence) and may improve hip (femoral neck) bone mineral density (standardised effect size 0.09, 95% CI − 0.03 to 0.21, 14 trials, low-certainty evidence). Higher doses of physical activity and programs involving multiple exercise types or resistance exercise appear to be most effective. Typical programs for which significant intervention impacts were detected in trials were undertaken for 60+ mins, 2–3 times/week for 7+ months. Observational studies suggested a positive association between long-term total and planned physical activity on bone health.

**Conclusions:**

Physical activity probably plays a role in the prevention of osteoporosis. The level of evidence is higher for effects of physical activity on lumbar spine bone mineral density than for hip. Higher dose programs and those involving multiple exercises and resistance exercises appear to be more effective.

**Supplementary Information:**

**Supplementary information** accompanies this paper at 10.1186/s12966-020-01040-4.

## Background

Osteoporosis is a major public health problem and is characterised by micro-architectural deterioration of bone tissue and low bone mineral density (BMD) which leads to reduced bone strength, increased bone fragility and a consequent increase in risk of skeletal fractures [1, 2]. Osteoporosis is known as a ‘silent disease’ as it is frequently undiagnosed until a symptomatic fracture occurs - usually at an older age [3]. The most common clinical manifestations of osteoporosis are fractures of the hip, vertebrae or wrist, with incidence increasing with age. Osteoporotic fractures are responsible for excess morbidity, mortality, reduction in quality of life, institutionalization and economic costs [1, 4–7]. For instance, in the UK it is estimated that fragility fractures cost the NHS £4.4 billion per year [8] and in the USA osteoporosis cost US$57 billion in 2018 with this figure projected to grow to over US$95 billion yearly by 2040 [9]. In light of worldwide increases in life expectation as well as the burden placed by osteoporosis fractures on societies, health systems and individuals, effective osteoporosis prevention strategies are essential.

Low bone mass is recognised as an important risk factor for fracture and therefore, a key target for osteoporosis prevention [1]. It is thought that disuse and inactivity generates unloading of the skeletal system resulting in reduced bone mass. Conversely, physical activity is thought to stimulate bone growth and preserve bone mass. Physical activity is an umbrella term that includes leisure time physical activity (exercise, sport), activities of daily living, household tasks and work [10]. The benefits of physical activity for healthy ageing are well established [11, 12] and various physical activity interventions for prevention and treatment of osteoporosis have been designed and evaluated. Various guidelines recommend engagement in physical activity for the management of osteoporosis [1], including for older people [13], however the effects of such interventions in older people who have not been diagnosed with osteoporosis i.e., in osteoporosis prevention have not been summarised. A summary of the evidence in this field is crucially important to enable specific recommendations on physical activity engagement for osteoporosis prevention to be made.

Previous reviews investigating the association between physical activity and osteoporosis prevention have only focused on specific types of physical activity, such as exercise [14–18], walking [19, 20], or sport [21], single body parts [22], male [15, 23] or female [14, 17, 20], and most were not specifically focused on older people or prevention. To address this evidence gap, and provide a comprehensive summary of the evidence in the field, we conducted a review investigating the effect of physical activity for prevention of osteoporosis in older people [24]. Given the worldwide low levels of physical activity, particularly pronounced in older people [25], a summary of the evidence on the effects of physical activity on the prevention of osteoporosis is important to inform public health initiatives and planning.

This review aimed to investigate the association between physical activity and osteoporosis prevention in older people (aged 65 years and above). The questions were: i) What is the association between physical activity and osteoporosis prevention in older people (> 64 years old)? ii) Is there a dose response association (volume, duration, frequency, intensity) between physical activity and prevention of osteoporosis? iii) Does the association vary by type or domain of physical activity? The focus was on primary prevention studies i.e., studies in the general community rather than studies in those with existing osteoporosis.

## Methods

We conducted a systematic review investigating the association between physical activity and osteoporosis prevention in older people. This review was commissioned by the World Health Organization (WHO) to assist the Guideline Development Group (GDG) develop the guidelines on physical activity and sedentary behaviour (2020) [26, 27]. It was submitted to the GDG for their consideration as they formulated their recommendations. The GDG decided on the scope of the guideline, the PICO (Population, Intervention, Comparison, Outcome) question, and the search strategy. The GDG initially requested an umbrella review (review of reviews). However, since no eligible reviews were found we included individual studies that were reported in the reviews identified by the search for reviews conducted in PubMed. To ensure that important studies were not missed, we conducted an additional search for individual studies and reviews after submission of the report. This manuscript includes the initial WHO report results as well as the expanded search results. We followed the preferred reporting items for systematic reviews and meta-analyses (PRISMA) guidelines [28, 29], and the PRISMA study flow diagram was used to document the screening process.

### Data source and search

A search for existing systematic reviews was conducted in PubMed for reviews published from 2008 up to November 2019 (Additional file 1, A). An expanded search was conducted in PubMed for individual studies published from January 2010 to March 2020 (Additional file 1, B). A second expanded search was conducted in PubMed and three additional databases (CINAHL, Embase, SPORTDiscus) for reviews published from 2008 up to July 2020 (Additional file 1, C).

### Study selection

Two reviewers screened all titles and abstracts to identify studies that addressed the present research questions. The full text of each study that potentially met the inclusion criteria was obtained and independently assessed for eligibility by two reviewers. Any disagreements were discussed and when consensus could not be reached, the eligibility of the study was decided following discussion with a third reviewer. We also searched for additional studies in the reference lists of eligible papers and relevant systematic reviews known by the team. All studies were selected according to eligibility criteria below and additional details on eligibility criteria can be found in Additional file 1, D.

#### Population

We included studies investigating adults aged 65 years and older. Studies that included younger participants were included if the mean age minus one standard deviation was more than 64 years and/or if participants met the age criteria at follow-up. Studies that recruited participants on the basis of having osteoporosis at baseline were excluded. We followed the WHO definition of osteoporosis on the basis of BMD measurement relative to reference values of young adults of the same sex [2]. No restriction was applied to participants’ health status or setting.

#### Exposure

The exposure of interest was any volume, duration, frequency, or intensity of physical activity. Studies where participants received multiple interventions were only included if the only difference between the groups was the physical activity intervention. We excluded studies that only used physical activity as a confounding variable as well as studies of multimodal interventions where physical activity was not the main component, or that did not present data on physical activity separately.

#### Comparison

We included studies that had no physical activity or lesser volume, duration, frequency, or intensity of physical activity as a comparator.

#### Outcome

Our outcome of interest was osteoporosis, including but not limited to BMD from any location (e.g., neck of femur, spine), bone mineral content (BMC), calcium bone index, cortical bone density, and bone quality index. We excluded studies that had fracture as an outcome in the absence of a bone mass measure.

#### Study design

We initially searched for systematic reviews and meta-analyses. Since we did not find any eligible systematic review, we identified reviews that included potentially eligible studies and screened all potential studies against our questions. The expanded search was targeted at individual studies that could have been missed by the initial search for reviews. We included individual studies (instead of reviews) that had the following study designs: randomised controlled trials, quasi-randomised controlled trials, prospective cohort studies, and retrospective cohort studies. We excluded cross-sectional and before-and-after studies.

We only included studies published with full-text in English and published in peer-reviewed journals. We excluded grey literature, including unpublished data, abstracts, and conference proceedings.

### Data extraction and quality assessment

One reviewer extracted information into standardised forms and a second reviewer checked all data. We extracted quantitative estimates for all outcome measures relevant to osteoporosis reported by the included studies.

#### Physical activity classification

We used the Prevention of Falls Network Europe (ProFaNE) taxonomy to classify the physical activity and exercise programs in the included trials (Additional file 2) [30]. The programs were classified as primarily involving the following exercise categories: i) gait, balance, coordination and functional task training (referred to as ‘balance and functional exercises’ for simplicity); ii) strength/resistance training (including power training; using resistance so referred to as ‘resistance exercises’); iii) flexibility; iv) three-dimensional (3D) exercise (with Tai Chi or dance subcategories); v) general physical activity (e.g., walking programs); vi) endurance; vii) other kinds of exercise. The taxonomy allows for more than one type of exercise to be delivered within a program. We also considered whether the exercise explicitly included bone loading (e.g., hopping or heel drops) and included this category (i.e., bone loading) as “other kinds of exercise”.

#### Quality assessment

We assessed the methodological quality of the randomised controlled trials and quasi-randomised trials using the PEDro scale with total scores ranging from 0 to 10 [31, 32]. We assessed the methodological quality of observational studies using a modified version of the Quality in Prognosis Studies (QUIPS) tool [33] adapted to studies of risk factors. The tool contains six domains and each is categorised as low, moderate or high risk of bias based on explicit criteria (Additional file 3). Overall risk of bias was considered ‘low’ if four or more domains (including study confounding) were rated as low risk of bias; otherwise, the overall risk of bias was considered ‘high’. Two reviewers assessed the risk of bias independently; discrepancies were resolved by a third reviewer.

Using the Grading of Recommendations Assessment, Development and Evaluation (GRADE) framework [34], we examined the quality of primary research and assessed the overall quality of evidence as ‘high’, ‘moderate’, ‘low’ or ‘very low’ in terms of presence and extent of four factors: risk of bias, inconsistency, imprecision, and publication bias. We did not consider the indirectness criterion because we only included similar studies in terms of population, intervention, comparator and outcome [35]. The quality of the evidence was rated for each outcome. Briefly, we downgraded the evidence by one level for limitation of study design if > 50% of included trials had a PEDro score < 6/10 [36]. We downgraded the evidence for imprecision if the total number of participants was less than 400 across all studies included in the meta-analysis [37]. We considered the results inconsistent if the heterogeneity between trials was large (I^2^ > 50%) or if there was wide variation of point estimates across the included studies [38]. We assessed publication bias (small study effect) by visual inspection of funnel plots and by performing a sensitivity analysis where we excluded studies with a small sample size (< 50 participants) and we considered whether their removal impacted the pooling of results [39].

#### Data synthesis and analysis

We pooled data from all relevant randomised controlled trials comparing physical activity with a control group for the main outcome of each trial. We also performed two additional analyses according to the two most commonly reported outcomes across the included studies. Within each analysis we performed subgroup analyses according to the physical activity classification, as per ProFaNE taxonomy. When data were available for more than one time-point, we extracted data from the time point closest to the end of the intervention. Mean estimates were extracted in the following hierarchical order: mean difference, change score and final score [40]. Where a trial included more than one intervention group, we included each intervention in a separate comparison and divided the number of participants in the control group accordingly to avoid double counting participants in the analyses [40]. We did not include the quasi-randomised trials and the trials investigating clinical populations in the meta-analysis.

We calculated the standardised mean difference (Hedges’ g) and 95% confidence interval (CI) and used random effects meta-analysis models as we considered that a range of true effects was likely but also undertook sensitivity analyses using fixed effect models. Hedges’ g was calculated using a combination of data format including mean difference, pre- and post score or change score data (as per individual study’s availability) and was standardised using the post-test score standard deviation where available. We used Comprehensive Meta-Analysis (Version 3, Biostat, Englewood NJ).

We undertook meta-regression to investigate the impact of different doses and types of physical activity interventions and study quality using Stata *metan* and *metareg* commands (Version 15, College Station, TX). For meta-regression we classified programs with 7800 total minutes (i.e., 150 mins × 52 weeks) or more as high dose programs. Type of physical activity intervention was coded according to the presence of ProFaNE taxonomy categories outlined above: balance/function, bone loading, resistance, multiple exercise and combination of multiple and resistance exercise types. We explored the impact of study methodological quality by undertaking meta-regression to compare effects in trials with PEDro scores equal or greater than 6 or below 6.

## Results

### Initial search

**Fig. 1:**
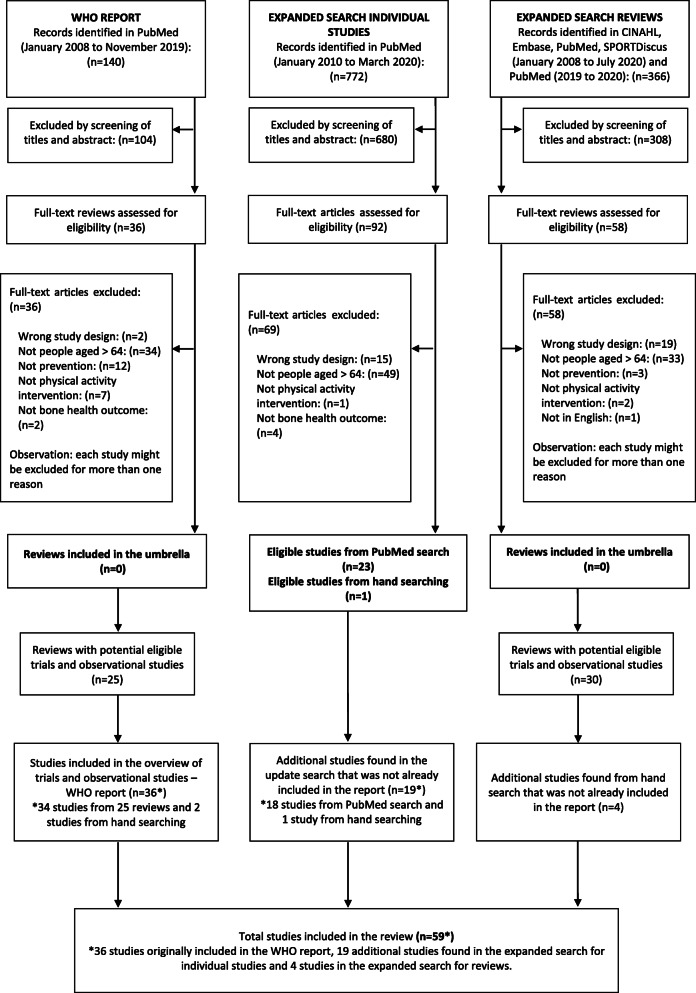
Flow chart of studies investigating physical activity and osteoporosis prevention in older people included in the WHO report (left size), in the expanded search for individual studies (middle) and expanded search for systematic reviews (right side)

The initial search for systematic reviews and meta-analyses did not identify sufficient evidence to answer the review questions. We screened the full texts of 36 reviews and no eligible reviews were found (Fig. 1). The main reasons for exclusion were reviews including younger participants (*n* = 34), participants with osteoporosis at baseline (management instead of prevention, *n* = 12), and not investigating whole body physical activity (e.g., whole body vibration, *n* = 7).

Amongst the 36 reviews which had their full text screened, 25 reviews included potentially eligible studies and their full texts were identified and assessed by two reviewers. We used the same eligibility criteria, but no restriction was applied for publication year of individual studies. We found 36 studies (trials and observational studies) investigating the association between physical activity and prevention of osteoporosis (34 identified from the reviews and 2 from hand searching) [41–76].

### Expanded search for individual studies

The expanded search yielded 772 records and the full texts of 92 records were screened (Fig. 1). A total of 24 studies met the eligibility criteria, 23 identified via PubMed search and one via hand searching. Out of the 24 studies identified, five had already been included in the WHO report [42, 47, 66, 68, 71]. Therefore, the expanded search found 19 additional studies [77–95].

### Expanded search for systematic reviews

The expanded search for systematic reviews identified a total of 366 reviews. We screened the full texts of 58 reviews and no eligible reviews were found (Fig. 1). Amongst the 58 reviews which had their full text screened, 30 reviews included potentially eligible individual studies and after assessing their full text we included 4 additional studies [96–99], resulting in a total of 59 studies included in this manuscript. The included studies were published between 1980 and 2020. There were 39 randomised controlled trials, 8 quasi-randomised trials and 12 observational studies (8 prospective and 4 retrospective studies). There were three cases where results from the same study were reported across multiple articles [53, 77, 81, 94, 96], all of which were included in this review as they reported results for different follow-up timepoints.

### Exposure

Within the included trials, 40 compared physical activity with a control intervention (Table 1); 11 compared two physical activity programs (Table 2); six trials (Table 3) and eight observational studies (Table 4) investigated different doses of physical activity. A total of 12 observational studies were included, seven investigated total physical activity, one leisure-time physical activity (exercise, transportation and sport), and five planned physical activity (four exercise and one sport-tennis) (Table 4).

**Table 1:** Description of included studies comparing physical activity with a control intervention

Reference PEDro score	Study design Allocated/Analysed	Participants (n, age mean (SD), % women, setting, health status)	Intervention Primary exercise type according to ProFANE^**a**^	Control^**b**^	Outcomes	Follow up (mo)	Results
Allison 2013 5/10 [41]	RCT 50/35	**Setting**: Community; United Kingdom **Health status**: Healthy **A. High impact exercise** ***n***= 50 (randomised); 35 (analysed) **Age**: 69.9 (4.0) **Female**: 0% **B. No exercise** Contralateral leg of each participant was used as control	**A.** High impact unilateral exercise programme (brief hopping exercise sessions) **Frequency**: 7 times/week **Intensity**: 5 sets of 10 multidirectional hops with a 15s rest period. Encouraged participants to continue to hop as high and as fast as they could. **Session duration**: ~15 min **Delivered by**: NR **Duration of the intervention (wks):** 52 **Primary exercise type**: Balance and function including bone loading (multidirectional hopping)	**B.** No exercises performed with the control leg	1. Femoral neck BMD 2. Trochanter BMD 3. Total hip BMD 4. Femoral neck BMC 5. Trochanter BMC 6. Total hip BMC	12	Final score (mean ± SD) 1. Femoral neck BMD^**c**^ A. Exercise: 0.954 ± 0.017 B. Control: 0.945 ± 0.018 2. Trochanter BMD A. Exercise: 0.923 ± 0.017 B. Control: 0.923 ± 0.018 3. Total hip BMD A. Exercise: 1.030 ± 0.017 B. Control: 1.027 ± 0.018 4.Femoral neck BMC^**c**^ A. Exercise: 5.54 ± 0.13 B. Control: 5.49 ± 0.14 5. Trochanter BMC A. Exercise: 16.45 ± 0.54 B. Control: 16.49 ± 0.57 6. Total hip BMC: A. Exercise: 40.49 ± 0.91 B. Control: 40.35 ± 0.97
^d^Armamento-Villareal 2012 7/10 [88]	RCT 107/107	**Setting**: Community; United States **Health status**: Obese older adults **A. Exercise group** ***n***= 26 **Age**: 70 (0.8) **Female**: 61% **B. Diet group (weight loss)** ***n***= 26 **Age**: 70 (0.8) **Female**: 65% **C. Diet and exercise** ***n***= 28 **Age**: 70 (0.8) **Female**: 57% **D. Control** ***n***= 27 **Age**: 69 (0.8) **Female**: 67%	**A. Exercise Group** involving aerobic exercises, progressive resistance training, and exercises to improve flexibility and balance. **Frequency**: 3 times/week **Intensity**: aerobic dance: from 65% of peak heart rate to 70%-85% of peak heart rate; resistance: from 1 to 2 sets at a resistance of approximately 65% of one-repetition maximum, with 8-12 reps to 2-3 sets at a resistance of 80% of one-repetition maximum, with 6 to 8 reps **Session duration:** 90 min **Delivered by**: Physical therapist **Duration of the intervention (wks)**: 52 **Primary exercise type**: Multiple (endurance plus flexibility plus resistance plus balance and function)	**D.** Control - no intervention or advice	1. Femoral neck BMD 2. Intertrochanter BMD 3. Femoral shaft BMD	12	Change (% ± SD) 1. Femoral neck BMD A. Exercise: 1.00 ± 0.76 B. Diet: -2.09 ± 1.07 C. Diet and exercise: -0.13 ± 0.91 D. Control: -0.08 ± 0.82 2. Intertrochanter BMD A. Exercise: 1.83 ± 0.59 B. Diet: -2.09 ± 1.06 C. Diet and exercise: -1.06 ± 0.98 D. Control: -0.18 ± 0.73 3. Femoral shaft BMD A. Exercise: 1.83 ± 0.59^**c**^ B. Diet: -2.47 ± 0.51 C. Diet and exercise: -0.92 ± 0.83 D. Control: 0.48 ± 0.61
Binder 2004 7/10 [43]	RCT 90/78	**Setting**: Hospital; home care programme and community; United States **Health Status**: People with a recent proximal femur fracture **A. Physical Therapy and exercise training** ***n***= 46 (randomised); 46 (analysed) **Age**: 80 (7) **Female**: 72% **B. Control - Home exercise** ***n***= 44 (randomised); 44 (analysed) **Age**: 81 (8) **Female**: 77%	**A.** Supervised physical therapy and exercise training involving flexibility, balance, coordination, movement speed and progressive resistance exercises. **Frequency:** 3 times/week **Intensity**: the resistance training started from 1-2 sets of 6-8 reps each exercise at 65% of 1RM; progressed to 8-12 reps 3 sets at 85%-100% of initial 1-RM. **Session duration**: 45 to 90 min **Delivered by**: Physical therapist **Duration of the intervention (wks)**: 26 **Primary exercise type**: Multiple (balance/function plus resistance)	**B.** Low-intensity home exercise programme	1. Whole body BMD 2. Hip BMD	6	Final score (mean ± SD) 1. Whole body BMD A. Physical therapy and exercise training: 1.03 ± 0.13 B. Home exercise programme: 1.00 ± 0.11 2. Hip BMD A. Physical therapy and exercise training: 0.64 ± 0.18 B. Home exercise programme: 0.69 ± 0.12 No significant group x time effects
Blumenthal 1991 6/10 [44]	RCT 101/84	**Setting**: NR; United States **Health status**: Healthy **A. Aerobic Training** ***n***= 33 (randomised) **B. Yoga and flexibility** ***n***= 34 (randomised) **C. Control** ***n***= 34 (randomised) **Age** (whole sample): 67 (range: 60-83) **Female**: NR	**A. Aerobic training:** Endurance training involving bicycle ergometry, brisk walking/jogging, and arm ergometry. **Frequency**: 3 times/week **Intensity**: 70% heart rate reserve **Session duration**: 60 min **Delivered by**: NR **Duration of intervention (wks):** 16 **Primary exercise type:** Endurance training **B. Yoga:** Supervised non-aerobic yoga programme. **Frequency**: at least 2 times/week **Intensity**: NR **Session duration**: 60 min **Delivered by:** NR **Duration of intervention (wks):** 16 **Primary exercise type**: Balance and function	**C**. Waiting list control: did not receive any form of treatment and were instructed not to change their physical activity habits and specifically not to engage in any aerobic exercise for the 4-month period.	1. Distal radius BMD (mg/ cm^2^)	4, 8, 14	1. Distal radius BMD: no between-group differences. Quantitative estimates not reported for between-group comparisons. Sub-analysis was performed comparing participants who decided to continue to exercise or not for men and women separately (randomisation was broken for this analysis): Female: Mean ± SD at eight months: Females who continued the programme: 0.7 ± 0.2 Females who discontinued the programme: 0.8 ± 0.1 Mean ± SD at fourteen months: Females who continued the programme: 0.7 ± 0.3 Females who discontinued the programme: 0.9 ± 0.2 Men Mean ± SD at eight months: Men who continued the programme: 1.2 ± 0.2 Men who discontinued the programme: 1.1 ± 0.2 Mean ± SD at fourteen months: Men who continued the programme: 1.4 ± 0.4 Men who discontinued the programme: 1.0 ± 0.3 Between-group difference: *p*<0.05
Bunout 2001 4/10 [45]	RCT 149/98	**Setting**: Outpatient clinic; Chile **Health Status**: Healthy **A. Resistance training** ***n***= 28 randomised; 16 (analysed) **Age**: 74.4 (3.3) **Female**: 75% **B. Control** ***n***= 31 (randomised); 25 (analysed) **Age**: 74.0 (3.7) **Female**: 48% **C. Supplementation + Resistance training** ***n***= 42 (randomised); 31 (analysed) **Age**: 73.7(3.0) **Female**: 40% **D. Supplementation** ***n***= 42 (randomised); 26 (analysed) **Age**: 74.7(3.7) **Female**: 62%	**A.** Endurance training consisting of exercise for upper and lower body, respiratory muscle training and walking. **Frequency**: 2 times/week **Intensity**: Graded by a coach using the Borg scale **Session duration**: 60 min **Delivered by**: Specialised coach **Duration of the intervention (wks)**: 78 **Primary exercise type**: Multiple (balance and function plus endurance)	**B.** No training	1. Whole body BMD 2. Whole body BMC	18	1. Whole body BMD Decreased significantly in all groups (*p* = 0.006), but the decline was less marked in the strength training combined with nutritional supplements compared with the other groups (statistically significant). Results reported in a graph and not possible to extract quantitative estimates. 2. Whole body BMC: NR
De Jong 2000 5/10 [46]	RCT 217/143	**Setting**: Community; Netherlands **Health status**: People with frailty and BMI ≤ 25 **A. Exercise** ***n***= 55 (randomised); 36 (analysed) **Age**: 76.5 (4.6) **Female**: 69% **B. Control** ***n***= 44 (randomised); 33 (analysed) **Age**: 78.8 (6.7) **Female**: 67% **C. Exercise + nutrition** ***n***= 60 (randomised); 39 (analysed) **Age**: 79.8 (5.8) **Female**: 74% **D. Nutrition** ***n***= 58 (randomised); 35 (analysed) **Age**: 79.6 (5.0) **Female**: 69%	**A.** Supervised group-based exercise programme involving muscle strength, coordination, flexibility, speed, endurance with use of ropes, weights and elastic bands. **Frequency**: 2 times/week **Intensity**: Moderate to high; 7 of a 10-point Borg scale **Session duration**: 45 min **Delivered by**: Skilled teachers and supervisor **Duration of the intervention (wks)**: 17 **Primary exercise type**: Balance and function	**B.** Social programme involving creative and social activities as well as educational sessions.	1. Whole body BMD	4.5	Change score (mean change ± SD) 1. Whole body BMD A. Exercise: 0.000 ± 0.022 B. Control: -0.003 ± 0.018 C. Combination group: 0.003 ± 0.023 D. Nutrition group: 0.006 ± 0.014 No between-group differences in the relevant comparisons to this review (ie, exercise vs control and combination vs nutrition)
Duckham 2015 6/10 [47]	RCT 319/283	**Setting**: General practice; United Kingdom **Health status**: Healthy **A. Home based exercise (OEP)** ***n***= 88 (randomised); 75 (analysed) **Age**: 71.4 (4.9) **Female**: 68% **B. Community based exercise (FaME)** ***n***= 105 (randomised); 94 (analysed) **Age**: 71.8 (5.5) **Female**: 60% **C. Control: Usual care** ***n***= 126 (randomised); 114 (analysed) **Age**: 72.2 (5.5) **Female**: 54%	**A. OEP**: Home exercise programme consisting of leg strengthening, balance exercise, and walking. **Frequency**: 3 sessions/week of home exercise; at least 2 sessions/week of walking **Intensity**: Walking moderate pace **Session duration:** 30 min/home exercise session, and 30 min/walking session **Delivered by**: Trial research staff in the one-off training **Duration of the intervention (wks)**: 24 **Primary exercise type**: Balance and function **B. FaME:** Falls and exercise management programme involving progressive resistance training, flexibility training, functional floor skill and adapted Tai Chi. Additionally, FaME intervention included home exercise based on EOP and walking. **Frequency**: 3-5 times/week [One exercise class, two home exercise session and at least two sessions of walking per week] **Intensity**: walking at moderate pace **Session duration**: 60 min/exercise class; 30 min/home exercise session; 30 min/walking session **Delivered by**: Postural stability instructor **Duration of the intervention (wks)**: 24 **Primary exercise type**: Balance and function	**C.** Usual care Participants not offered the FaME or OEP programmes	1. Femoral neck BMD 2. Trochanter BMD 3. Total hip BMD 4. Upper neck BMD 5. Lumbar spine BMD 6. Distal radius BMD 7. Whole body BMD 8. Whole body BMC	6	Mean difference (95% CI) 1. Femoral neck BMD A. OEP: -0.003 (-0.011 to 0.005) B. Community based: -0.002 (-0.010 to 0.005) 2. Trochanter BMD A. OEP: -0.005 (-0.032 to 0.022) B. Community based: 0.000 (-0.025 to 0.026) 3. Total hip BMD A. OEP: -0.008 (-0.034 to 0.019) B. Community based: 0.003 (-0.022 to 0.028) 4. Upper neck BMD A. OEP: 0.003 (-0.018 to 0.023) B. Community based: 0.006 (-0.013 to 0.026) 5. Lumbar spine BMD A. OEP: 0.003 (-0.012 to 0.019) B. Community based: 0.005 (-0.010 to 0.020) 6. Distal radius A. OEP: 0.001 (-0.008 to 0.010) B. Community based: -0.009 (-0.018 to -0.000)^**c**^ 7. Whole body BMD A. OEP: 0.003 (-0.002 to 0.008) B. Community based: -0.003 (-0.007 to 0.002) 8. Whole body BMC A. OEP: 0.8 (-22.0 to 23.6) B. Community based: -6.6 (-27.9 to 14.7)
Englund 2005 5/10 [48]	RCT 48/40	**Setting**: Community; Sweden **Health status**: Healthy **A. Exercise (COMB)** ***n***= 24 (randomised); 21 (analysed) **Age**: 72.8 (3.6) **B. Control** ***n***= 24 (randomised); 19 (analysed) **Age**:73.2 (4.9) **Female**: 100%	**A.** Supervised exercise programme involving a combination of strengthening, aerobic, balance and coordination exercises **Frequency**: 2 times/week **Intensity**: 2 sets of 8-12 reps (strengthening exercise) **Session duration**: 50 min **Delivered by**: Physiotherapist **Duration of the intervention (wks)**: 47 **Primary exercise type**: Multiple (balance and function plus resistance plus endurance)	**B.** No training	1. Lumbar Spine BMD 2. Femoral neck BMD 3. Trochanter BMD 4. Ward’s triangle BMD 5. Whole body BMD 6. Arms BMD 7. Whole body BMC	12	Mean difference (95% CI) (on % changes) 1. Lumbar spine BMD: 2.1 (-0.4 to 3.4) 2. Femoral neck BMD: 0 (-3.8 to 2.6) 3. Trochanter BMD: 3.4 (-1.2 to 7.3) 4. Ward's triangle BMD: 2.2 (1.8 to 12.9)^**c**^ 5. Whole body BMD: 0.1 (-1.3 to 2.2) 6. Arms BMD: 0 (-1.9 to 2.8) 7. Whole body BMC: 1.3 (-0.3 to 3.1)
Helge 2014 5/10 [50]	RCT 27/23	**Setting**: Community; Denmark **Health status**: Healthy **A. Football group** ***n***= 9 (randomised); 9 (analysed) **Age**: 68.0 (4.0) **B. Resistance training** ***n***= 9 (randomised); 8 (analysed) **Age**: 69.1 (3.1) **C. Control** ***n***= 8 (randomised); 6 (analysed) **Age**: 67.4 (2.7) **Female**: 0%	**A. Football group:** Supervised progressive football training **Frequency**: 1.7 (0.3) times/week (range: 1.2-2.2) **Intensity**: 82% of maximum heart rate (range 64 to 90%) **Session duration**: 45 to 60 min **Delivered by**: NR **Duration of the intervention (wks)**: 52 **Primary exercise type**: Balance and function (football) **B. Resistance training:** Progressive resistance training for core and upper and lower body **Frequency**: 1.9 (0.2) times/week (range: 1.4-2.2) **Intensity**: started from 3 sets of 16-20 RM to 4 sets of 8 RM **Session duration**: 45 to 60 min **Delivered by**: NR **Duration of the intervention (wks)**: 52 **Primary exercise type**: Resistance (seated)	**C.** Inactive control	1. Whole body BMD 2. Right femoral neck BMD 3. Left femoral neck BMD 4. Right femoral shaft BMD 5. Left femoral shaft BMD 6. Total right proximal femur BMD 7. Total left proximal femur BMD	12	Final score (mean ± SE) 1. Whole body BMD A. Football: 1.211 ± 0.036 B. Resistance: 1.225 ± 0.024 C. Control: 1.268 ± 0.030 2. Right femoral neck BMD A. Football: 0.921 ± 0.034 B. Resistance: 1.000 ± 0.042 C. Control: 1.008 ± 0.063 3. Left femoral neck BMD A. Football: 0.939 ± 0.034 B. Resistance: 1.006 ± 0.036 C. Control: 1.018 ± 0.043 4. Right femoral shaft BMD A. Football: 1.156 ± 0.042 B. Resistance: 1.229 ± 0.056 C. Control: 1.254 ± 0.059 5. Left femoral shaft BMD A. Football: 1.143 ± 0.043 B. Resistance: 1.229 ± 0.057 C. Control: 1.282 ± 0.045 6. Total right proximal femur BMD A. Football: 0.982 ± 0.031 B. Resistance: 1.066 ± 0.048 C. Control: 1.083 ± 0.048 7. Total left proximal femur BMD A. Football: 0.989 ± 0.031 B. Resistance: 1.069 ± 0.048 C. Control: 1.117 ± 0.041
Jessup 2003 5/10 [52]	RCT 18/16	**Setting**: Retirement Community; United States **Health Status**: Healthy **A. Multi-component intervention** ***n***= 9 (randomised); 8 (analysed) **Age**: 69.1 (2.8) **B. Control** ***n***= 9 (randomised); 8 (analysed) **Age**: 69.4 (4.2) **Female**: 100%	**A.** Supervised exercise programme involving resistance training, load-bearing walking with use of weights vest, stair-climbing, and balance training. **Frequency**: 3 times/week **Intensity**: 8-10 reps of 50% of 1RM, progressed to 75% of 1RM (resistance training **Session duration**: 60 to 90 min exercise training session; 30 to 45 min walking **Delivered by:** Co-investigator and/or research assistant **Duration of the intervention (wks)**: 32 weeks **Primary exercise type**: Multiple (balance and function plus resistance plus endurance)	**B.** Control	1. Femoral neck BMD 2. Lumbar spine BMD	8	Change score (ANCOVA, p-value) 1. Femoral neck BMD A. Exercise: 1.7 B. Control: -0.04 F (1, 15) = 7.38, *P*=0.016 2. Lumbar spine BMD A. Exercise: 0.11 B. Control: -0.003 F (1, 15) = 2.70, *P*=0.121 Final score (mean ± SD) 1. Femoral neck BMD A. Exercise: 0.74 ± 0.05 B. Control: 0.74 ± 0.13 2. Lumbar spine BMD A. Exercise: 0.88 ± 0.08 B. Control: 1.14 ± 0.32
Karinkanta 2007¶ 7/10 [53]	RCT 149/144	**Setting**: Community; Finland **Health Status**: healthy and excluded participants with osteoporosis **A. Balance-jumping training** ***n***= 37(randomised); 35 (analysed) **Age**: 72.9 (2.3) **B. Resistance training** ***n***= 37 (randomised); 37(analysed) **Age**: 72.7 (2.5) **C. Combined Balance-jumping and resistance training** ***n***= 38 (randomised); 36 (analysed) **Age**: 72.9 (2.2) **D. Control** ***n***= 37 (randomised); 36 (analysed); **Age**: 72.0 (2.1) **Female**: 100%	**A. Balance-jumping training:** Balance training including static and dynamic balance exercise, agility training, impact exercises and changes of direction exercise. **Intensity**: NR **Primary exercise type**: Balance and function including bone loading (jumps) **B. Resistance training:** Tailored progressive resistance training programme for large muscle groups. **Intensity**: Initially 2 sets of 10-15 reps at intensity 50-60% of 1RM, progressed to 3 sets of 8-10 reps at 75-80% of 1RM. Rate of perceived exertion: above 18 out of 20 **Primary exercise type**: Resistance **C. Combined Balance-jumping and resistance training**: A combination of A & B on alternate weeks. **Primary exercise type**: Multiple (balance and function plus resistance) For all exercise groups: **Frequency**: 3 times/week **Session duration**: 50 min **Delivered by:** Exercise leaders **Duration of the intervention (wks)**: 52	**D.** Control: maintain their pre-study level of physical activity during the 12-month trial	1. Femoral neck BMC 2. Distal tibia trabecular density (mg/cm^3^)	12	Final score (mean ± SD) 1. Femoral neck BMC A. Balance: 2.73 ± 0.40 B. Resistance: 2.71 ± 0.33 C. Combined: 2.65 ± 0.29 D. Control: 2.67 ± 0.44 2. Distal tibia trabecular density (mg/cm^3^) A. Balance: 224 ± 34 B. Resistance: 219 ± 26 C. Combined: 215 ± 39 D. Control: 226 ± 33
^e^Karinkanta 2009¶ 5/10 [98]	RCT 149/126	**Setting**: Community; Finland **Health Status**: Healthy and excluded participants with osteoporosis **A. Balance jumping training group** ***n***= 37 (randomised); 33 (analysed) **Age**: 72.9 (2.3) **B. Resistance training group** ***n***= 37 (randomised); 34 (analysed) **Age**: 72.7 (2.5) **C. Combined resistance and balance jumping training group** ***n***= 38 (randomised); 32 (analysed) **Age**: 72.9 (2.2) **D. Non-training control group** ***n***= 37 (randomised); 27(analysed) **Age**: 72.0 (2.1) **Female:** 100%	**A. Balance-jumping training:** Balance training (static and dynamic), agility training, impact exercises and changes of direction exercise. **Intensity**: NR **Primary exercise type**: Balance and function including bone loading (jumps) **B. Resistance training:** Tailored progressive resistance for large muscle groups. **Intensity**: Initially 2 sets of 10-15 reps at intensity 50-60% of 1RM, progressed to 3 sets of 8-10 reps at 75-80% of 1RM. Rate of perceived exertion: above 18 out of 20 **Primary exercise type**: Resistance **C. Combined Balance-jumping and resistance training**: A combination of A & B on alternate weeks. **Primary exercise type**: Multiple (balance and function plus resistance including bone loading) For all exercise groups: **Frequency**: 3 times/week **Session duration**: 50 min **Delivered by:** Exercise leaders **Duration of the intervention (wks)**: 52	**D.** Control: maintain their pre-study level of physical activity	1. Femoral neck section moduls (Z) (mm^3^) 2. Tibia midshaft desnity-weighted polar section modulus (BSI) (mm^3^)	24	% Mean difference compared to control (95% CI) 1. Femoral neck Z A. Balance: 3.6 (-0.8 to 8.2) B. Resistance: 3.5 (-0.8 to 8.1) C. Combined: 0.3 (-4.0 to 4.8) 2. Tibia midshaft BSI A. Balance: 0.2 (-1.1 to 1.6) B. Resistance: 0.3 (-1.0 to 1.6) C. Combined:0.6 (-0.7 to 1.9)
^d^Kemmler 2012§ 4/10 [94]	Quasi-randomised trial 137/85	**Setting**: Community; Germany **Health Status**: Osteopenia **A. Exercise group** ***n***= 86 (randomised); 41 (analysed) **Age**: 55.0 (3.4) **B. Control-no training** ***n***= 51 (randomised); 44 (analysed) **Age**: 55.8 (3.1) **Female**: 100%	**A.** Supervised group class that includes warm-up/ endurance, jumping and resistance exercise + home training that includes rope skipping, isometric exercises, elastic belt and stretching exercises **Frequency**: Supervised group classes: 2 times/week; home training 2 times/week (supervised group classes: 3 times/week; home training 1 time/week in the year 4 and 5) **Intensity**: Aerobic dance: 70% to 85% maximum heart rate and peak ground reaction forces (GRF) at approximately 3 to 4 times bodyweight; Multilateral jumping: 4 sets of 15 reps and GRF at approximately 4 times of bodyweight; Resistance: from 1 to 4 sets, 4 to 12 reps, 70% to 90% 1 RM (2 to 3 minute-rest) to 2 to 3 sets, 20 to 25 reps, 50% to 55% 1 RM (1 to 2-minute rest) **Session duration**: 60 to 65 min/ supervised group session; 20 min/home training session **Delivered by**: Certified trainers **Duration of intervention (wks):** 49 to 50 weeks/year throughout the 12 years **Primary exercise type**: Multiple (endurance plus resistance with bone loading)	**B.** No training: maintain own’s habitual lifestyle	1. Lumbar spine (L1-L4) BMD 2. Femoral neck BMD	144	Mean difference (95% CI) 1. Lumbar spine BMD 0.030 (0.011 to 0.049)^**c**^ 2. Femoral neck BMD 0.024 (0.009 to 0.039)^**c**^
^d^Kemmler 2016§ 4 /10 [93]	Quasi-randomised trial 137/67	**Setting**: Community; Germany **Health status**: Osteopenia **A. Exercise group** ***n***= 86 (randomised); 39 (analysed) **Age**: 55.0 (3.5) **B. Control-no training** ***n***= 51 (randomised); 28 (analysed) **Age**: 56.0 (3.0) **Female**: 100%	**A.** Supervised group class (aerobic dance exercise, jumping and resistance exercise) + Home training (rope skipping, isometric and dynamic resistance exercise and stretching/ flexibility exercise) five months after study started **Frequency**: Year 4 and 5: supervised group classes: 3 times/week; home training 1 time/week All other years: supervised group classes: 2 times/week; home training 2 times/week **Intensity**: Aerobic dance: 70% to 85% maximum heart rate and 2 to 3 bodyweight peak ground reaction forces (GRF) Multilateral jumping: 4 sets of 15 reps at GRF of 3 to 4.5 bodyweightResistance exercise: from 1 to 4 sets of 4 to 12 reps at intensity of 70% to 90% 1 RM (2- to 3-minute rest) to 2 to 3 sets of 20 to 25 reps at an intensity of 50% to 55% 1 RM (1- to 2-minute rest) **Session duration**: 60 to 65 min/ supervised group session; 20 to 25 min/home training session **Delivered by**: NR **Duration of intervention (wks):** 49 to 50 weeks/year throughout the 16 years **Primary exercise type:** Multiple (endurance plus resistance with bone loading)	**B.** No training – maintain present lifestyle	1. Lumbar spine BMD 2. Total hip BMD	192	Absolute mean difference between groups (95% CI) 1. Lumbar spine BMD Period 1 (baseline to year 4): 2.37 (0.97 to 3.77)^**c**^ Period 2 (year 5 to year 8): 0.81 (0.15 to -1.76) Period 3 (year 8 to year 12): 0.78 (0.03 to -1.58) Period 4 (year 12 to year 16): 0.75 (0.12 to 1.38) ^**c**^ 2. Total hip BMD Period 1 (baseline to year 4): 0.92 (0.24 to -2.08) Period 2 (year 5 to year 8): 0.81 (0.12 to 1.92)^**c**^ Period 3 (year 8 to year 12): 0.16 (0.59 to -0.91) Period 4 (year 12 to year 16): 1.15 (0.08 to 2.22) ^**c**^
^d^Kim 2018 6 /10 [95]	Pilot RCT 51/41	**Setting**: Outpatient department of a hospital; South Korea **Health status**: Diagnosis of Stage I to III prostate cancer receiving androgen deprivation therapy without osteoporosis **A. Home-based exercise intervention for preventing osteoporosis (HEPO) intervention** ***n***= 26 (randomised); 23 (analysed) **Age**: 70.5 (5.0) **B. Control-stretching exercise (STR)** ***n***= 25 (randomised); 18 (analysed) **Age**: 71.0 (5.5) **Female:** 0%	**A.** Home-based exercise (HEPO)**.** A core program (weight-bearing exercise and resistance exercise) + optional program (stabilization/ balance exercise and circuit resistive calisthenics). Two 30-minute education sessions with a workbook preceded the start of the exercise and ten 15-minute sessions of telephone counselling **Frequency**: 3 to 5 times/week **Intensity**: The weight-bearing goal involved at least 150 minutes per week of moderate-intensity work, starting at an intensity of 11 to 12 on the rate of perceived exertion scale and increasing for 6 months to 13 to 15. The resistance exercise protocol started at free weight and gradually increased to loads of 10% of body weight. **Session duration**: ~40 min **Delivered by**: Exercise physiologist **Duration of the intervention (wks):** 24 **Primary exercise type**: Resistance with bone loading	**B.** Whole body stretching exercise (STR)	1. Lumbar spine (L1-L4) BMD 2. Femoral neck BMD 3. Total hip BMD	6	Change score (mean ± SD) 1. Lumbar spine (L1-L4) BMD A. HEPO: -0.027 ± 0.007 B. STR: -0.031 ± 0.008 2. Femoral neck BMD A. HEPO: -0.014 ± 0.007 B. STR: -0.015 ± 0.008 3. Total hip BMD A. HEPO: -0.008 ± 0.006 B. STR: -0.011 ± 0.006
Kohrt 1997 3/10 [55]	Quasi-randomised trial 39/30	**Setting:** NR; United States **Health Status**: Healthy **A. Ground reaction forces training** ***n***= 14 (randomised); 12 (analysed) **Age**: 66.0 (1.0) **B. Joint reaction forces training** ***n***= 13 (randomised); 9 (analysed) **Age**: 65.0 (1.0) **C. Control** ***n***= 12 (randomised); 9 (analysed) **Age**: 68.0 (1.0) **Female**: 100%	**A. Ground reaction forces training:** Individualised exercise training focusing on activities that involved ground-reaction forces, such as walking, jogging and/or stair climbing. **Frequency**: 3 to 5 times/week **Intensity**: 60-70% to 80-85% maximum heart rate **Session duration:** 30-45 minutes/day **Delivered by**: NR **Duration of the intervention (wks)**: 36 **Primary exercise type**: Multiple (balance and function plus endurance plus flexibility) **B. Joint reaction forces training:** Individualised exercise training including activities that involved joint-reaction forces, such as weightlifting and rowing. **Frequency**: 3 to 5 sessions/week **Intensity**: Weightlifting: 2-3 sets of 8-12 reps; Rowing: 60-70% to 80-85% of maximum heart rate **Session duration:** NR for the total session duration; however; rowing took 15 to 20 min **Delivered by**: NR **Duration of the intervention (wks)**: 36 **Primary exercise type**: Multiple (resistance plus endurance plus flexibility)	**C.** No exercise	1. Whole body BMD 2. Lumbar spine L2–L4 BMD 3. Femoral neck BMD 4. Trochanter BMD 5. Ward’s BMD 6. Ultra-distal wrist BMD 7. One-third distal wrist BMD	12	Between-group analysis relative to control 1. Whole body BMD A. Ground reaction: *p* < 0.05 B. Joint reaction: *p* < 0.01 2. Lumbar spine L2–L4 BMD A. Ground reaction: *p* < 0.05 B. Joint reaction: *p* < 0.01 3. Femoral neck BMD A. Ground reaction: *p* < 0.01 B. Joint reaction: no difference 4. Trochanter BMD A. Ground reaction: no difference B. Joint reaction: no difference 5. Ward’s BMD A. Ground reaction: *p* < 0.01 B. Joint reaction: *p* < 0.05 6. Ultra-distal wrist BMD A. Ground reaction: no difference B. Joint reaction: no difference 7. One-third distal wrist BMD A. Ground reaction: no difference B. Joint reaction: no difference Quantitative estimates were not reported (chance scores are provided in a graph)
^d^Korpelainen 2010‡ 7/10 [96]	RCT 160/100	**Setting**: Community; Finland **Health status**: Women with hip and radius osteopenia **A. Exercise group** ***n***= 84 (randomised); 55 (analysed) **Age**: 72.7 (1.1) **B. Control group** ***n***= 76 (randomised); 45 (analysed) **Age**: 72.6 (1.2) **Female**: 100%	**A.** Supervised balance, leg strength, and impact training and home exercise **Frequency**: 1 time/week of training session; 1 time/day of home exercise training **Intensity**: NR **Session duration:** 60 min/ supervised session, and 20 min/ home exercise following program **Delivered by**: Physical therapist **Duration of the intervention**: 24 weeks/year **Primary exercise type**: Multiple (balance and function plus resistance with bone loading)	**B.** Control	1. Femoral neck BMD 2. Trochanter BMD 3. Total proximal femur BMD 4. Femoral neck BMC 5. Trochanter BMC 6. Total proximal femur BMC	48, 60, 72	Mean difference (95% CI) 1. Femoral neck BMD At 4 year: 0.01 (-0.02 to 0.03) At 5 year: 0.01 (-0.03 to 0.02) At 6 year: 0.00 (-0.02 to 0.02) 2. Trochanter BMD At 4 year: 0.01 (-0.02 to 0.03) At 5 year: 0.01 (-0.02 to 0.03) At 6 year: 0.01 (-0.02 to 0.04) 3. Total proximal femur BMD At 4 year: 0.01 (-0.01 to 0.04) At 5 year: 0.01 (-0.02 to 0.03) At 6 year: 0.01 (-0.01 to 0.04) 4. Femoral neck BMC At 4 year: -0.01 (-0.14 to 0.11) At 5 year: -0.03 (-0.16 to 0.09) At 6 year: -0.01 (-0.13 to 0.11) 5. Trochanter BMC At 4 year: -0.22 (-0.87 to 0.23) At 5 year: -0.30 (-0.51 to 0.60) At 6 year: -0.25 (-0.78 to 0.33) 6. Total proximal femur BMC At 4 year: 0.01 (-1.56 to 0.76) At 5 year: 0.01 (-1.72 to 0.74) At 6 year: 0.01 (-1.68 to 0.81)
^d^Korpelainen 2006‡ 6/10 [79]	RCT 160/136	**Setting**: Community; Finland **Health status**: Women with hip and radius osteopenia **A. Exercise group** ***n***= 84 (randomised); 69(analysed) **Age**: 72.9 (1.1) **B. Control group** ***n***= 76 (randomised); 67 (analysed) **Age**: 72.8 (1.2) **Female**: 100%	**A.** Supervised balance, jumping, and impact group training **Frequency**: 1 time/week of training session; 1 time/day of home exercise training **Intensity**: NR **Session duration:** 60 min/training session, and 20 min/home training **Delivered by**: Physical therapist **Duration of the intervention (wks)**: 24 weeks/year [exercise took place at home for other times during the year and in total there were 72 weeks supervised group exercise] for 30 months. **Primary exercise type**: Multiple (balance and function plus resistance with bone loading)	**B.** Control	1. Femoral neck BMD 2. Trochanter BMD 3. Total proximal femur BMD 4. Femoral neck BMC 5. Trochanter BMC 6. Total proximal femur BMC 7. Distal radius BMD 8. Ultradistal radius BMD	30	Mean difference (95% CI) 1. Femoral neck BMD 0.007 (-0.010 to 0.024) 2. Trochanter BMD 0.011 (-0.014 to 0.035) 3. Total proximal femur BMD 0.004 (-0.021 to 0.030) 4. Femoral neck BMC -0.018 (-0.134 to 0.100) 5. Trochanter BMC 0.043 (-0.514 to 0.600)^**c**^ 6. Total proximal femur BMC -0.332 (-1.433 to 0.769) 7. Distal radius BMD -0.003 (-0.017 to 0.011) 8. Ultradistal radius BMD -0.004 (-0.018 to 0.008)
Kwon 2008 3/10 [56]	Quasi-randomised trial 40/NR	**Setting:** Community; Korea **Health status:** Healthy **A. Multicomponent intervention** ***n***= 20 (randomised) **Age**: 77.4 (2.56) **B. Control** ***n***= 20 (randomised) **Age**: 77.0 (3.33) **Female**: 100%	**A.** Combined training programme consisting of aerobic exercise, resistance training (free weights) and balance exercise. **Frequency:** 3 times/week **Intensity**: Aerobic exercises: started with 40-55% and up to 65-75% heart rate reserve; Resistance exercise: 8-12 reps at 75% of 1RM **Session duration:** 60 min **Delivered by:** NR **Duration of the intervention (wks)**: 24 **Primary exercise type**: Multiple (balance and function plus endurance plus resistance)	**B.** Control	1. Whole body BMD 2. Lumbar (L2-L4) spine BMD 3. Femoral neck BMD 4. Ward’s triangle BMD 5. Greater trochanter BMD	6	Final score (mean ± SD) 1. Whole body BMD A. Exercise: 0.92 ± 0.07 B. Control: 0.88 ± 0.05 2. Lumbar (L2-L4) spine BMD A. Exercise: 0.85 ± 0.15 B. Control: 0.85 ± 0.10 3. Femoral neck BMD A. Exercise:0.68 ± 0.12 B. Control: 0.70 ± 0.07 4. Ward’s triangle BMD A. Exercise: 0.48 ± 0.10 B. Control: 0.46 ± 0.08 5. Greater trochanter BMD^**c**^ A. Exercise: 0.59 ± 0.05 B. Control: 0.58 ± 0.12
Lau 1992 4/10 [57]	RCT 60/50	**Setting**: Hostel; Hong Kong **Health Status**: Healthy **A. Exercise group and placebo calcium supplementation** ***n***= 11 (analysed) **Age**: mean age (range): 79 (76-81) **B. Calcium supplementation** ***n***= 12 (analysed) **Age**: mean age(range): 75 (72-79) **C. Calcium supplementation and exercise** ***n***= 15 (analysed) **Age**: mean age(range): 76 (73-80) **D. Control** ***n***= 12 (analysed) **Age**: mean age (range): 75 years (71-78) **Female**: 100%	**A.** Supervised exercise involving moving the upper trunk while standing. **Frequency**: 4 times/week **Intensity**: Submaximal exertion effort **Session duration**: 15 min **Delivered by**: NR **Duration of the intervention (wks)**: 40 **Primary exercise type**: Balance and function	Control	1. Femoral neck BMD 2. Wards triangle BMD 3.Intertrochanteric area BMD 4. Lumbar spine (L2-L4) BMD	10	Change score (%; mean, 95% CI) 1. Femoral neck BMD A. Exercise: -6.6 (-12 to 0.8) B. Control: -1.1 (-7.4 to 5.3) C. Supplement: -3.5 (-9 to 1.8) D. Supplement and exercise: 5.0 (-0.77 to 10) 2. Wards triangle BMD A. Exercise: -6.0 (-15 to 3.2) B. Control: -2.4 (-10 to 5.9) C. Supplement: 2.5 (-5.9 to 11) D. Supplement and exercise: 17 (3 to 31) 3.Intertrochanteric area BMD A. Exercise: 0.1 (-6.5 to 6.7) B. Control: 0.25 (-3.3 to 3.8) C. Supplement: 2 (-1.6 to 5.7) D. Supplement and exercise: 11 (1.3 to 22) 4. Lumbar spine BMD A. Exercise: -1.9 (-6.7 to 2.8) B. Control: -2.5 (-6.5 to 1.4) C. Supplement: -0.08 (-5.2 to 5.1) D. Supplement and exercise: -1.1 (-3.7 to 1.4)
Lord 1996 4/10 [58]	RCT 179/138	**Setting**: Community, Australia **Health Status**: Healthy **A. Multicomponent exercise** ***n***= 90 (randomised); 68 (analysed) **Age**: 71.7 (5.4) **B. Control** ***n***= 89 (randomised); 70 (analysed) **Age**: 71.5 (5.3) **Female**: 100%	**A.** Supervised group-based exercise programme involving aerobic exercise, balance training, strengthening exercise, and stretching. **Frequency**: 2 times/week **Intensity**: NR **Session duration**: 60 min **Delivered by**: Instructors trained to provide the programme **Duration of the intervention (wks)**: 52 (only 42 weeks for exercise as there were breaks in between) **Primary exercise type**: Balance and function	No exercise	1. Femoral neck BMD 2. Trochanter BMD 3. Lumbar spine (L2-L4) BMD	12	Final score (mean ± SD) / Change score (mean % change ± SD) 1. Femoral neck BMD A. Exercise: 0.791 ± 0.122 / 1.52 ± 5.19 B. Control: 0.776 ± 0.110 / 3.12 ± 6.52 2. Trochanter BMD A. Exercise: 0.707 ± 0.127 / 0.69 ± 4,64 B. Control: 0.672 ± 0.123 / 0.73 ± 5.28 3. Lumbar spine (L2-L4) BMD A. Exercise: 1.036 ± 0.209 / 1.07 ± 2.59 B. Control: 1.008 ± 0.189 / 0.36 ± 3.91
Marques 2011 5/10 [59]	RCT 60/60	**Setting**: Community; Portugal **Health Status**: Healthy **A. Multi-component training** ***n***= 30 (randomised and analysed) **Age**: 70.1 (5.4) **B. Control** ***n***= 30 (randomised and analysed) **Age**: 68.2 (5.7) **Female**: 100%	**A.** Progressive multicomponent exercise training consisting of moderate to high impact weight-bearing activities, endurance, balance exercise, and agility training. **Frequency**: 2 times/week **Intensity**: Stepping exercise: at 120-125 beats/min. Weight bearing and strength exercise: from 2 sets of 8 reps to 3 sets of 15 reps **Session duration:** 60 min **Delivered by:** Physical education instructors specialised in physical activity for older adults **Duration of the intervention (wks)**: 32 **Primary exercise type**: Balance and function with bone loading (heel drops)	**B.** Control	1. Femoral neck BMD 2. Total femur BMD 3. Trochanter BMD 4. Intertrochanteric BMD 5. Lumbar spine (L1-L4) BMD	8	Final score (mean ± SD) 1. Femoral neck BMD^**c**^ A. Exercise: 0.717 ± 0.085 B. Control: 0.671 ± 0.051 2. Total femur BMD A. Exercise: 0.832 ± 0.104 B. Control: 0.823 ± 0.058 3. Trochanter BMD A. Exercise: 0.628 ± 0.081 B. Control: 0.628 ± 0.034 4. Intertrochanteric BMD A. Exercise: 0.989 ± 0.148 B. Control: 0.977 ± 0.075 5. Lumbar spine (L1-L4) BMD A. Exercise: 0.868 ± 0.094 B. Control: 0.863 ± 0.065
McCartney 1995 3/10 [60]	RCT 68/NR	**Setting**: NR; Canada **Health status**: Healthy **A. Exercise** ***n***= 37 (randomised) **Age**: 73 (3) **Female**: 54% **B. Control** ***n***= 31(randomised) **Age**: 72 (3) **Female**: 74%	**A.** Progressive resistance training for upper and lower body, and abdominals. Completed in as a circuit. **Frequency**: 2 sessions/week **Intensity**: 2 sets of each exercise at 50% of 1RM to 3 sets of 80% 1RM **Session duration:** NR **Delivered by:** NR **Duration of the intervention (wks)**: 42 weeks **Primary exercise type**: Resistance	**B**. Control Offered a supervised walking programme. **Frequency:** 2 sessions/week **Intensity**: low **Session duration:** NR **Delivered by:** NR **Duration of the intervention (wks)**: 42 weeks	1. Lumbar spine (L2-4) BMD 2. Whole body BMD 3. Lumbar spine (L2-4) BMC 4. Whole body BMC	10.5	No significant changes in BMD and BMC as a result of the training programme. Quantitative estimates not reported.
McMurdo 1997 4/10 [61]	RCT 118/92	**Setting**: Community; United Kingdom **Health status**: Healthy **A. Exercise and calcium supplementation** ***n***= 44 (analysed) **B. Calcium supplementation** ***n***= 48 (analysed) **Age**: 64.5 (range 60-73) **Female**: 100%	**A.** Exercise programme involving weight bearing exercise to music and calcium supplementation (1000 mg calcium daily, as calcium carbonate) **Frequency**: 3 times/week **Intensity**: NR **Session duration**: 45 min **Delivered by:** NR **Duration of the intervention (wks)**: 30 (three 10-week terms) **Primary exercise type**: Balance and function	**B.** Taking calcium supplementation (1000 mg calcium daily, as calcium carbonate)	1. Lumbar BMD 2. Distal forearm (non-dominant) BMC 3. Ultra distal forearm (non-dominant) BMC	24	Change score (mean % change ± 95% CI) 1. Lumbar BMD A. Exercise and calcium: -0.91 (-6.8 to 5.0) B. Calcium: -2.65 (-5.7 to 0.4) 2. Distal forearm (non-dominant) BMC A. Exercise and calcium: -2.18 (-3.0 to -1.4) B. Calcium: -1.38 (-2.2 to -0.6) 3. Ultra distal forearm BMC^**c**^ A. Exercise and calcium: 1.14 (-0.8 to 3.1) B. Calcium: -2.6 (-4.6 to -0.6)
^e^Nichols 1995 4/10 [99]	RCT 34/28	**Setting**: Community; United States **Health status**: Healthy and active **A. Weight training group** ***n***= 17(randomised); 9 (analysed at 12-month) **Age**: 67.8 (standard error: 1.6) **B. Control** ***n***= 17(randomised); 8 (analysed at 12-month) **Age**: 65.2 (1.2) **Female**: 100%	**A.** Supervised, isotonic training (leg flexion and extension, back extension, trunk flexion, bench press, latissimus dorsi pull-down, shoulder press and seated row) **Frequency**: 3 times/week **Intensity**: Commenced with one set of 10-12 reps at an intensity of 50% of 1RM and progressed to three sets at 80% of 1RM from third week **Session duration**: NR **Delivered by**: NR **Duration of the intervention (wks)**: 52 **Primary exercise type**: Resistance	**B.** Contunue current endurance exercise program	1. Lumbar spine (L2-4) BMD2. Femoral neck BMD 3. Trochanter BMD 4. Total body BMD	12	Final score (mean ± SE) 1. Lumnar spine BMD A. Weight training: 1.025 ± 0.04 B. Control: 1.012 ± 0.03 2. Femoral neck BMD A. Weight training: 0.776 ± 0.03 B. Control: 0.772 ± 0.02 3. Trochanter BMD A. Weight training: 0.670 ± 0.02 B. Control: 0.666 ± 0.03 4. Total body BMD A. Weight training: 0.976 ± 0.02 B. Control: 0.979 ± 0.03
Paillard 2004 5/10 [62]	RCT 21/21	**Setting**: Community; France **Health status**: Healthy **A. Walking group** ***n***= 11 (randomised and analysed) **Age**: 65.5 (2) **B. Control** ***n***= 10 (randomised and analysed) **Age**: 66.8 (2) **Female**: 0%	**A.** Individualised brisk walking programme **Frequency**: 5 times/week **Intensity**: Lactate threshold (minimum heart rate: 131 beats/minute; maximum heart rate: 156 beats/minute) **Session duration**: 45 to 60 min **Delivered by**: NR **Duration of the intervention (wks)**: 12 **Primary exercise type**: Endurance (walking)	**B.** Control	1. Hip BMD 2. Whole body BMD	3	Final score (mean ± SD) 1. Hip BMD A. Walking: 0.84 ± 0.11 B. Control: 0.95 ± 0.12 2. Whole body BMD A. Walking: 1.06 ± 0.11 B. Control: 1.02 ± 0.13
Park 2008 5/10 [63]	RCT 50/50	**Setting**: Community; Korea **Health status**: Healthy **A. Multi-component training** ***n***= 25 (randomised and analysed) **Age**: 68.3 (3.6) **B. Control** ***n***= 25 (randomised and analysed) **Age**: 68.4 (3.4) **Female**: 100%	**A.** Exercise training including stretching, strength training, weight-bearing exercise, balance and posture correction training. **Frequency**: 3 times/week **Intensity**: 65%-75% of the maximum heart rate **Session duration**: 60 min **Delivered by**: NR **Duration of the intervention (wks)**: 48 **Primary exercise type**: Multiple balance/ function plus endurance (weight-bearing)	**B.** Control	1. Femoral neck BMD 2. Ward’s triangle BMD 3. Trochanter BMD 4. Lumbar spine (L2 to L4) BMD	12	Final score (mean ± SD) 1. Femoral neck BMD^**c**^ A. Exercise: 0.857 ± 0.078 B. Control: 0.748 ± 0.063 2. Ward’s triangle BMD A. Exercise: 0.659 ± 0.086 B. Control: 0.576 ± 0.079 3. Trochanter BMD^**c**^ A. Exercise: 0.725 ± 0.081 B. Control: 0.677 ± 0.062 4. Lumbar spine (L2 to L4) BMD A. Exercise: 1.059 ± 0.082 B. Control: 0.891 ± 0.155
Pruitt 1995 4/10 [64]	RCT 40/26	**Setting**: Community; America **Healthy status**: Healthy **A. High intensity resistance training** ***n***= 15 (randomised); 8 (analysed) **Age**: 67.0 (0.5) **B. Low intensity resistance training** ***n***= 13 (randomised); 7 (analysed) **Age**: 67.6 (1.4) **C. Control** ***n***= 12 (randomised); 11 (analysed) **Age**: 69.6 (4.2) **Female**: 100%	**A. High intensity resistance training:** High intensity supervised resistance training comprising exercises for upper and lower extremities with the use of equipment. **Intensity:** 2 sets of 7 reps at 80% 1RM **Primary exercise type**: Resistance **B. Low intensity resistance training:** Supervised resistance training comprising exercises for upper and lower extremities using equipment. **Intensity:** 3 sets of 14 reps at 40% 1RM For both A and B: **Frequency**: 3 times/week **Session duration**: 50 to 55 min **Delivered by**: NR **Duration of the intervention (wks)**: 52 **Primary exercise type**: Resistance	**C.** No training	1. Total hip BMD 2. Femoral neck BMD 3. Ward’s triangle BMD 4. Lumbar spine (L2-L4) BMD	12	Change score (mean ± SD) 1. Total hip BMD A. High intensity: 0.005 ± 0.014 B. Low intensity: 0.008 ± 0.012 C. Control: 0.007 ± 0.010 2. Femoral neck BMD A. High intensity: -0.002 ± 0.154 B. Low intensity: 0.025 ± 0.008 C. Control: 0.005 ± 0.019 3. Ward’s triangle BMD A. High intensity: 0.018 ± 0.032 B. Low intensity: 0.022 ± 0.045 C. Control: 0.008 ± 0.036 4. Lumbar spine (L2-L4) BMD A. High intensity: 0.007 ± 0.018 B. Low intensity: 0.005 ± 0.027 C. Control: 0.000 ± 0.020
Rhodes 2000 5/10 [65]	RCT 44/38	**Setting**: Community; Canada **Health Status**: Healthy **A. Resistance training** ***n***= 22 (randomised); 20 (analysed) **Age**: 68.8 (3.2) **B. Control** ***n***= 22 (randomised); 18 (analysed) **Age**: 68.2 (3.5) **Female**: 100%	**A**. Supervised progressive resistance training comprising exercises for large muscle groups. **Frequency**: 3 times/week **Intensity:** 3 sets of 8 reps at 75 % 1 RM **Session duration**: 60 min **Delivered by**: Professional lifestyle and fitness consultants **Duration of the intervention (wks)**: 52 **Primary exercise type**: Resistance	**B.** No exercise programme and were instructed to maintain their normal lifestyle throughout the study duration.	1. Femoral neck BMD 2. Ward’s triangle BMD 3. Trochanter BMD 4. Lumbar spine (L2-L4) BMD 5. Femoral neck BMC 6. Ward’s triangle BMC 7. Trochanter BMC 8. Lumbar spine (L2-L4) BMC	12	Final score (mean ± SD) 1. Femoral neck BMD A. Exercise: 0.83 ± 0.12 B. Control: 0.73 ± 0.10 2. Ward’s triangle BMD A. Exercise: 0.70 ± 0.11 B. Control: 0.59 ± 0.12 3. Trochanter BMD A. Exercise: 0.75 ± 0.11 B. Control: 0.67 ± 0.11 4. Lumbar spine (L2-L4) BMD A. Exercise: 1.13 ± 0.18 B. Control: 1.01 ± 0.17 5. Femoral neck BMC A. Exercise: 4.02 ± 0.22 B. Control: 3.48 ± 0.19 6. Ward’s triangle BMC A. Exercise: 1.85 ± 0.19 B. Control: 1.51 ± 0.18 7. Trochanter BMC A. Exercise: 9.04 ± 0.33 B. Control: 8.83 ± 0.36 8. Lumbar spine (L2-L4) BMC A. Exercise: 45.86 ± 2.7 B. Control: 42.50 ± 2.6
Rikli 1990 1/10 [67]	Quasi-randomised trial 37/31	**Setting**: Local retirement community; United States **Health Status**: Healthy **A. General exercise** ***n***= 13 (randomised); 10 (analysed) **Age**: 72.2 (5.57) **B. General exercise + weight** ***n***= 13 (randomised); 10 (analysed) **Age**: 71.6 (5.66) **C. Control** ***n***= 11 (randomised); 11 (analysed) **Age**: 70.8 (8.43) **Female**: 100%	**A. General exercise:** Group-based aerobic exercise training for large muscle groups. **Frequency**: 3 times/week **Intensity**: 60-70% maximum heart rate **Session duration**: 30 to 50 min **Delivered by**: NR **Duration of the intervention (wks)**: 40 **Primary exercise type:** endurance **B. General exercise + weight:** Group-based aerobic exercise training plus upper body progressive resistance training. The resistance training was performed without supervision. **Frequency**: 3 times/week **Intensity**: 60-70% maximum heart rate for aerobic activities **Session duration**: 50 to 70 min **Delivered by**: Assistants **Duration of the intervention (wks)**: 40 **Primary exercise type**: Multiple resistance plus endurance	**C**. No exercise	1. Distal radius BMC/BW 2. Distal radius BMC	10	Change score (%) 1. Distal radius BMC/BW^**c**^ A. General exercise: 0.921 B. General exercise and weight: 1.734 C. Control: -2.577 2. Distal radius BMC^**c**^ A. General exercise: 1.023 B. General exercise and weight: 1.743 C. Control: -2.499
Sakai 2010 4/10 [68]	RCT 94/84	**Setting**: Community, Japan **Health Status**: Healthy **A. Exercise** ***n***= 49 (randomised); 47 (analysed) **Age**: 68.3 (0.8) **B. Control** ***n***= 45 (randomised); 37 (analysed) **Age**: 68.2 (0.5) **Female**: 100%	**A.** Home balance exercises involving unipedal standing exercise with their eyes open (single leg standing) **Frequency**: 3 sets/day; 7 days/week **Intensity**: NA **Session duration**: 2 min/set **Delivered by**: NA (home exercise) **Duration of the intervention (wks)**: 26 **Primary exercise type**: Balance/function	**B.** Usual activity	1. Femoral neck BMD 2. Trochanter BMD 3. Intertrochanter BMD 4. Ward's triangle BMD 5. Total hip BMD	6	% mean difference (p-value) 1. Femoral neck: *p*=0.993 2. Trochanter: *p*=0.801 3. Intertrochanter: *p*=0.968 4. Ward’s triangle *p*=0.096 5. Total hip: *p*=0.889 Change score reported in a graph
Smith 1981 2/10 [70]	Quasi-randomised trial 80/51	**Setting**: Nursing home; United States **Health Status**: Healthy **A. Physical activity group + placebo tablets** ***n***= 19 (randomised); 12 (analysed) Age: 82.9 (6.1) **B. Control (placebo tablet)** ***n***= 26 (randomised); 18 (analysed) **Age**: 81.9 (7.4) **C. Calcium and vitamin D** ***n***= 17 (randomised); 10 (analysed) **Age**: 80.7 (4.8) **D. Physical activity + calcium and vitamin D** ***n***= 18 (randomised); 11 (analysed) **Age**: 84.3 (5.1) **Female**: 100%	**A.** Light-to-mild seated exercises including sideward leg spread, leg walk, running in place, arm cross, sideward bend and chair pull. **Frequency**: 3 times/week **Intensity**: 70% of the sampled VO_2_ max **Session duration**:30 min **Delivered by**: NR **Duration of the intervention (wks)**: 156 **Primary exercise type**: Endurance (seated)	**B.** Placebo tablets Received 360 mg of lactose, 5mg of magnesium stearate and 80 mg of microcrystalline cellulose in the placebo tablets	1. Radius BMC	36	Change score (%) Radius BMC^**c**^ A. Exercise: 2.29% D. Control: - 3.29%
^e^Snow 2000 3/10 [100]	Quasi-randomised trial 18/18	**Setting**: Community; United States **Health status**: Healthy **A. Exercise group** ***n***= 9 (randomised and analysed) **Age**: 66.4 (1.7) **B. Control** ***n***= 9 (randomised and analysed) **Age**: 61.8 (2.5) **Female**:100%	**A**. Year1: 9-month training (10 min of warm-up, 35 min of lower-body resistance training, including stepping, squats, chair raises, forward lunges, lateral lunges and toe raises, using the weighted vest, 10-15 min of cool-down activities. Jumping exercises were included during the fourth months of training without the weighted vests Year 2-5 emphasied maintenance in lower body exercises utilising the weighted vest included more jumps per class than year 1 and encouraged use of the weighted vest while jumping. **Frequency**: 3 times/week **Intensity:** 3 to 5 sets of 10 to 15 reps using the weight vest. Vest resistance set from 5% of body weight and was gradually increased (approximaltey 1 to 2% every 2 weeks) until 10% of body weight; Beyond 10% of body weight, resistance was increased at 0.5% to 1% every 2 weeks. **Session duration**: 60 **Delivered by**: NR **Duration of the intervention (wks)**: 260 **Primary exercise type**: Resistance with bone loading (jumping)	**B.** Maintain and record their physical activity during the study and did not engage in weighted vest of jumping activity	1. Femoral neck BMD 2. Trochanter BMD 3. Total hip BMD	60	% change (mean ± SE) 1. Femoral neck BMD^**c**^ A. Exercise group: 1.54 ± 2.37 B. Control: -4.43 ± 0.93 2. Trochanter BMD^**c**^ A. Exercise group: -0.24 ± 1.02 B. Control: -3.43% ± 1.09 3. Total hip BMD^**c**^ A. Exercise group: -0.82 ± 1.04 B. Control: -3.80% ± 1.03%
Taaffe 1996 4/10 [101]	RCT 36/21	**Setting**: Community; United States **Health status**: Healthy **A. High intensity resistance training group** ***n***= 12 (randomised); 7 (analysed) **Age**: 67.0 (0.2) **B. Low intensity resistance training group** ***n***= 13 (randomised); 7 (analysed) **Age**: 67.6 (0.5) **C. Control** ***n***= 11 (randomised); 7 (analysed) **Age**: 69.6 (1.3) **Female**: 100%	Supervised exercise training targeted thigh muscle strength including leg press, knee extension and knee flexion. Exercise sessions were bracketed by warm up and cool-down periods **A. Intensity**: 1 set of 14 reps at an intensity of 40% 1RM and 2 sets of 7 reps at an intensity of 80% of 1RM **B. Intensity:** 3 sets of 14 reps at an intensity of 40% of 1RM For both groups **Frequency**: 3 times/week **Session duration**: NR **Delivered by**: NR **Duration of the intervention (wks)**: 52 **Primary exercise type**: Resistance	**D**. Control	1. Middle third of the femur BMD 2. Thigh BMD	12	Change score (mean % ± SEM) 1. Middle third of the femur BMD^**c**^ A. High intensity: 1.0± 1.0 B. Low inteisty: -2.2 ± 0.5 C. Control: -1.8 ± 0.6 2. NR
Taaffe 1999 5/10 [72]	RCT 53/46	**Setting**: Community; United States **Health status**: Healthy **A. High-intensity resistance training (1 day per week)** ***n***= 14 (randomised); 11 (analysed) **Age**: 68.5 (3.6) **Female**: 36% **B. High-intensity resistance training (2 days per week)** ***n***= 14 (randomised); 12 (analysed) **Age**: 69.4 (3.0) **Female**: 29% **C. High-intensity resistance training (3 days per week**) ***n***= 11 (randomised & analysed) **Age**: 71.0 (4.1) **Female**: 36% **D. Control** ***n***= 14 (randomised); 12 (analysed) **Age**: 68.9 (3.6) **Female**: 43%	**A, B & C.** Supervised resistance training targeting the major upper and lower body muscle groups. **A. Frequency**: 1 time/week **B. Frequency**: 2 times/week **C. Frequency**: 3 times/week **Intensity**: Started at 60% of the 1RM and gradually increase in intensity to 3 sets of 8 reps at 80% of 1 RM **Session duration**: NR **Delivered by**: NR **Duration of the intervention (wks)**: 24 **Primary exercise type**: Resistance	**D**. Control	1. Lumbar spine (L2-L4) BMD 2. Total hip BMD 3. Midradius BMD 4. Total body BMC	6	Final score (mean ± SEM) 1. Lumbar spine (L2-L4) BMD A. Resistance 1x/week: 1.025 ± 0.006 B. Resistance 2x/week: 1.033 ± 0.006 C. Resistance 3x/week: 1.032 ± 0.007 D. Control: 1.041 ± 0.006 2. Total hip BMD A. Resistance 1x/week: 0.865 ± 0.010 B. Resistance 2x/week: 0.866 ± 0.006 C. Resistance 3x/week: 0.864 ± 0.010 D. Control: 0.873 ± 0.010 3. Midradius BMD A. Resistance 1x/week: 0.605 ± 0.003 B. Resistance 2x/week: 0.604 ± 0.003 C. Resistance 3x/week: 0.608 ± 0.003 D. Control: 0.601 ± 0.003 4. Total body BMC A. Resistance 1x/week: 2552 ± 15 B. Resistance 2x/week: 2530 ± 14 C. Resistance 3x/week: 2525 ± 14 D. Control: 2536 ± 14
Villareal 2003 4/10 [73]	Quasi-randomised trial 28/28	**Setting**: Community; United States **Health status**: Mild to moderate physical frailty on hormone replacement therapy **A. Supervised multi-component training** ***n***= 14 (randomised and analysed) **Age**: 81 (3) **B. Control: Home exercise** ***n***= 14 (randomised and analysed) **Age**: 81 (3) **Female**: 100%	**A.** Supervised exercise programme involving flexibility and balance exercises, resistance training and endurance exercises. **Frequency**: 3 times/week **Intensity**: Resistance training: from 1-2 sets of 8-12 reps at 65% of 1RM to 2-3 sets of 6-8reps at 75–85% of 1RM Endurance: from 65–75 to 85–90% peak heart rate **Session duration**: 90 to 120 min **Delivered by**: Exercise physiologists **Duration of the intervention (wks)**: 36 weeks **Primary exercise type**: Multiple (resistance plus balance/function plus endurance)	**B**. Home exercise programme focusing on flexibility **Frequency**: 2-3 times/week	1. Total hip BMD 2. Femoral neck BMD 3. Trochanter BMD 4. Lumbar spine BMD 5. Whole body BMD	9	Change score (mean ± SD) 1. Total hip BMD A. Exercise: 0.003 ± 0.011 B. Control: 0.009 ± 0.011 4. Lumbar spine BMD^**c**^ A. Exercise: 0.034 ± 0.022 B. Control: 0.015 ± 0.022 5. Whole body BMD A. Exercise: 0.015 ± 0.015 B. Control: 0.002 ± 0.015 No quantitative estimates reported for: 2. Femoral neck BMD 3. Trochanter BMD (There were no significant group-by-time interaction effects) Results reported in a graph
Villareal 2004 4/10 [74]	RCT 119/112	**Setting**: Community; United States **Health status**: Mild-to-moderate physical frailty **A. Exercise training (ET)** ***n***= 69 (randomised); 65 (analysed) **Age**: 83 (4) **Female**: 52% **B. Home exercise (HOME)** ***n***= 50 (randomised); 47 (analysed) **Age**: 83 (4) **Female**: 55%	**A.** Supervised exercise programme involving flexibility and balance exercises, resistance training and endurance exercises. **Frequency**: NR **Intensity:** Resistance training: started from 1-2 sets of 6-8 reps at 65-75% of 1RM to 3 sets of 8-12 reps at 85-100% 1RM Endurance training*:* started from 15 mins at 65-75% of peak heart rate to 30 mins at 85-90% of peak heart rate **Session duration**: NR **Delivered by**: NR **Duration of the intervention (wks)**: 36 **Primary exercise type**: Multiple (resistance plus balance/function plus endurance)	**B.** Home exercise programme focusing on flexibility	1. Total hip BMD 2. Femoral neck BMD 3. Trochanter BMD 4. Lumbar spine (L2-L4) BMD 5. Whole Body BMD	9	Final score (mean ± SD) 1. Total hip BMD A. Exercise: 0.85 ± 0.19 B. Control: 0.75 ± 0.15 2. Femoral neck BMD A. Exercise: 0.70 ± 0.17 B. Control: 0.63 ± 0.11 3. Trochanter BMD A. Exercise: 0.65 ± 0.17 B. Control: 0.58 ± 0.12 4. Lumbar spine (L2-L4) BMD A. Exercise: 1.08 ± 0.28 B. Control: 0.97 ± 0.23 5. Whole Body BMD A. Exercise: 1.09 ± 0.18 B. Control: 1.03 ± 0.17
von Stengel 2011 7/10 [78]	RCT 151/141	**Setting**: Community; Germany **Health Status**: Healthy **A. Conventional multicomponent training** ***n***= 50 (randomised); 47 (analysed) **Age**: 68.6 (3) **B. Wellness control** ***n***= 51 (randomised); 48 (analysed) **Age**: 68.1 (2.7) **Female**: 100%	**A**. Training sessions consisting of aerobic dancing; progressive coordination and balance training; functional gymnastics and isometric strength training; and progressive upper body exercises. Additionally, participants were requested to carry out a home training session. **Frequency**: 4 times/week [i.e. 2 controlled training session/week; 2 home exercise/week] **Intensity**: Dance aerobic: 70–80% maximum heart rate; Functional gymnastics and isometric strength: 6-10s of maximum exertion and 20-30s of active rest; Upper body exercise: 3 sets 15 reps **Session duration:** 60 min/training session; 20 min/home training session **Delivered by**: Certified instructors **Duration of the intervention (wks)**: 72 **Primary exercise type**: Multiple (balance/function, plus flexibility plus resistance plus endurance (dance)	**B:** Low intensity wellness programme that includes light physical exercises and a relaxation programme **Frequency**: 1 time/week **Intensity**: Light **Session duration**: NR **Delivered by**: NR **Duration of the intervention (wks)**: 72 (10 weeks of training were intermitted by a break of 10 weeks and the training cycle was repeated throughout 72 weeks)	1. Total hip BMD 2. Lumbar spine (L1-L4) BMD	18	Mean difference (95% CI): 1. Total hip BMD: 0.002 (-0.007 to 0.012) 2. Lumbar spine: 0.015 (0.001 to 0.029)^**c**^
^d^Winters-Stone 2014 7/10 [97]	RCT 51/43	**Setting**: Community; United States **Health Status**: Prostate cancer survivors receiving androgen deprivation therapy without osteoporosis **Age:** 70.2 **A. Progressive, moderate-intensity resistance + impact training (POWIR)** **n**=29 (randomised); 24(analysed) **B. Control- flexibility training (FLEX)** ***n***= 22 (randomised); 12 (analysed) **Female:** 0%	**A.** Progressive, moderate-intensity resistance for upper and lower body + impact training consisted of two footed jumps from the group to a target height 1” from the floor with a bent-knee landing with weighted vests **Frequency:** 3 times/week (two supervised classes + one home-based session) **Intensity:** Lower body training 1-2 sets of 8-12 reps from 0% to 15% of the body weight. Upper body training started from 1-2 sets of 12-14 reps at 13-15 RM to 1-2 sets of 8 to 10 reps at 8-10RM. Jumping started from 1-4 sets of 10 reps at 0-1% body weight to 9 to 10 sets of 10 reps at 10% body weight using weighted vest **Session duration**: 60 min **Delivered by**: Trained exercise instructors **Duration of the intervention (wks)**: 52 **Primary exercise type**: Resistance with bone loading	**B.** FLEX Control: Whole body stretching and relaxation	1. Lumbar spine (L1-L4) BMD 2. Total hip BMD 3. Greater trochanter BMD 4. Femoral neck BMD	12	Final score (mean ± SD) 1. Lumbar spine BMD A. POWIR: 1.123 ± 0.241 B. FLEX: 1.094 ± 0.156 2. Total hip BMD A. POWIR: 0.956 ± 0.135 B. FLEX: 0.971 ± 0.129 3. Greater trochanter BMD A. POWIR: 0.776 ± 0.131 B. FLEX: 0.783 ± 0.112 4. Femoral neck BMD A. POWIR: 0.752 ± 0.131 B. FLEX: 0.791 ± 0.098
Woo 2007 6/10 [75]	RCT 180/176	**Setting**: Community; Hong Kong **Heath status**: Healthy **A. Tai Chi** **n**=60 (randomised); 58 (randomised) **Age**: 68.2 years **B. Resistance training** ***n***= 60 (randomised); 59 (randomised) **Age**: 68.7 years **C. No Treatment** ***n***= 60 (randomised); 59 (randomised) **Age**: 68.1 years **Female**: 50%	**A. Tai Chi:** 24-forms of Tai Chi using Yang style **Frequency:** 3 times/week **Intensity**: NR **Session duration**: NR **Delivered by**: NR **Duration of the intervention (wks)**: 52 weeks **Primary exercise type**: 3D (Tai Chi) **B. Resistance training:** Resistance training with the use of medium strength TheraBand **Frequency:** 3 times/week **Intensity**: 30 times with medium strength TheraBand **Session duration**: NR **Delivered by**: NR **Duration of the intervention (wks)**: 52 weeks **Primary exercise type**: Resistance	**C.** No intervention	1. Total hip BMD 2. Total spine BMD	12	Change score (mean % change ± SE) Men 1. Total hip BMD A. Tai Chi: -0.48 ± 0.37 B. Resistance: -1.20 ± 0.38 C. Control: -0.15 ± 0.38 2. Total spine BMD A. Tai Chi: 1.35 ± 0.40 B. Resistance: 1.27 ± 0.42 C. Control: 0.54 ± 0.42 Women 1. Total hip BMD A. Tai Chi: 0.07 ± 0.64^**c**^ B. Resistance: 0.09 ± 0.62^**c**^ C. Control: -2.25 ± 0.60 2. Total spine BMD A. Tai Chi: 0.10 ± 0.50 B. Resistance: 1.98 ± 0.48 C. Control: 0.98 ± 0.47
Yoo 2010 4/10 [76]	RCT 28/21	**Setting**: Community; Korea **Health status**: Healthy **A. Exercise** ***n***=14 (randomised); 11 (analysed) **Age**: 70.9 (2.7) **B. Control** ***n***= 14 (randomised); 10 (analysed) **Age**: 71.1 (2.7) **Female**: 100%	**A**. Supervised walking exercise programme involving walking ankle weights. **Frequency**: 3 times/week **Intensity**: Maintained at 60% of heart rate reserve **Session duration**: 60 min **Delivered by**: NR **Duration of the intervention (wks)**: 12 **Primary exercise type**: Endurance (walking with ankle weights)	**B**. Control	1. Femoral neck BMD 2. Femoral Ward’s BMD 3. Femoral trochanter BMD 4. Spine BMD 5. Whole body BMD	3	Final score (mean ± SD) 1. Femoral neck BMD A. Exercise: 0.770 ± 0.132 B. Control: 0.729 ± 0.124 2. Femoral Ward’s BMD A. Exercise: 0.580 ± 0.158 B. Control: 0.584 ± 0.164 3. Femoral trochanter BMD A. Exercise: 0.708 ± 0.105 B. Control: 0.687 ± 0.136 4. Spine BMD A. Exercise: 1.056 ± 0.188 B. Control: 1.010 ± 0.167 5. Whole body BMD A. Exercise: 1.057 ± 0.077 B. Control: 1.028 ± 0.109

*BMC* bone mineral content (g), *BMD* bone mineral density (g/cm^2^ unless specified), *BMI* body mass index (kg/m^2^), *BW* bone width, *NR* not reported, *RCT* randomised controlled trial. In studies where other groups or other outcomes not of interest to this study were included (example supplement, or whole-body vibration) we only included and extracted information for the groups and for the comparisons that were relevant to this study (i.e., those where the effect of physical activity could be evaluated). When data was available for more than one time-point, we extracted the post-intervention data and any additional follow-up. Mean estimates were extracted in the following hierarchical order: mean difference, change score and final score

^a^Exercise is a physical activity that is planned, structured and repetitive and aims to improve or maintain physical fitness. There is a wide range of possible types of exercise, and exercise programmes often include one or more types of exercise. We categorised exercise based on a modification of the Prevention of Falls Network Europe (ProFaNE) taxonomy that classifies exercise type as: i) gait, balance, and functional training; ii) strength/ resistance (including power); iii) flexibility; iv) three- dimensional (3D) exercise (e.g., Tai Chi, Qigong, dance); v) general physical activity; vi) endurance; and vii) other kind of exercises. The taxonomy allows for more than one type of exercise to be delivered within a programme. We also considered whether the exercise explicitly included bone loading eg hopping or heel drops

^b^A control intervention is one that is not thought to improve bone health, such as general health education, social visits, very gentle exercise, or ’sham’ exercise not expected to impact on bone health.

^**c**^indicates statistically significant between-group differences at *p* < 0.05

^d^indicates studies that were found in the expanded search for individual studies conducted in March 2020 in PubMed

^e^indicates studies that were found in the updated search for systematic reviews conducted in July 2020 in PubMed, Embase, CINAHL, SPORTDiscus

§ and ‡ and ¶ indicate articles reporting results from the same study

**Table 2:** Description of included studies comparing two or more forms of physical activity

Reference PEDro score	Study design Allocated/Analysed	Participants (n, age mean (SD), % women, setting, health status)	Intervention Primary exercise type according to ProFANE^**a**^	Relevant comparison	Outcomes	Follow up (mo)	Results
^d^Armamento-Villareal 2020 7/10 [87]	RCT 160/141	**Setting**: Community; United States **Health status**: Obese older adults **A. Aerobic exercise** ***n***= 40 (randomised); 35 (analysed) **Age**: 70 (4) **Female**: 65% **B. Resistance exercise** ***n***= 40 (randomised); 35 (analysed) **Age**: 70 (5) **Female**: 63% **C. Combination** ***n***= 40 (randomised); 35 (analysed) 40 **Age**: 70 (5) **Female**: 60% **D. Control** [control group not relevant for this review question]	**A. Aerobic exercise** **Frequency**: 3 times/week **Intensity**: ~65% of peak heart rate, gradually increased to 70% to 85%. **Session duration**: ~60 min **Delivered by**: Exercise physiologists **Duration of the intervention (wks):** 26 **Primary exercise type**: Endurance **B. Resistance exercise:** nine upper-body and lower-body exercises using weightlifting machines **Frequency**: 3 times/week **Intensity**: 1 to 2 sets of 8 to 12 reps at 65% of the 1 RM and increased progressively to 2 to 3 sets at ~85% of the 1-RM. **Session duration**: ~60 min **Delivered by**: Exercise physiologists **Duration of the intervention (wks):** 26 **Primary exercise type**: Resistance **C. Combination:** Aerobic and resistance exercise training sessions **Frequency**: 3 times/week **Intensity**: Aerobic exercise: ~65% of their peak heart rate, gradually increased to 70% to 85%. Resistance exercise: 1 to 2 sets of 8 to 12 reps at 65% of the 1 RM and increased progressively to 2 to 3 sets at ~85% of the 1-RM **Session duration**: 75 to 90 min **Delivered by**: Exercise physiologists **Duration of the intervention (wks):** 26 **Primary exercise type**: Multiple (endurance plus resistance)	A vs B A vs C B vs C	1. Total hip BMD 2. Femoral neck BMD 3. Trochanter BMD 4. Intertrochanter BMD 5. Lumbar spine BMD 6. Whole body BMD 7. One-third radius BMD	6	Change score (mean ± SD) 1. Total hip BMD A. Aerobic: -0.027 ± 0.004^¥^ B. Resistance: -0.006 ± 0.004 C. Combination: -0.012 ± 0.004 2. Femoral neck BMD A. Aerobic: -0.020 ± 0.003^¥^ B. Resistance: -0.003 ± 0.003 C. Combination: -0.008 ± 0.003 3. Trochanter BMD A. Aerobic: -0.035 ± 0.007^¥^ B. Resistance: -0.006 ± 0.007 C. Combination: -0.016 ± 0.007 4. Intertrochanter A. Aerobic: -0.035 ± 0.007^¥^ B. Resistance: -0.006 ± 0.007 C. Combination: -0.016 ± 0.007 5. Lumbar spine BMD A. Aerobic: 0.002 ± 0.006 B. Resistance: 0.008 ± 0.006 C. Combination: 0.008 ± 0.005 6. Whole body BMD A. Aerobic: -0.003 ± 0.005 B. Resistance: 0.005 ± 0.005 C. Combination: 0.002 ± 0.005 7. One-third radius BMD A. Aerobic: -0.001 ± 0.001 B. Resistance: -0.0020 ± 0.001 C. Combination: -0.001 ± 0.002
Ashe 2013 [77]	RCT 155/135	**Setting**: Community; Canada **Health status**: Healthy **A. Balance and tone (BT)** ***n***= 49 (randomised); 42 (analysed) **Age**: 69.9 (3.1) **B. Once a week resistance training (RT1)** ***n***= 54 (randomised); 47 (analysed) **Age**: 69.4 (3.0) **C. Twice a week resistance training (RT2)** ***n***= 52 (randomised); 46 (analysed) **Age**: 69.2 (3.0) **Female**: 100%	**A. BT:** Group-based supervised intervention consisting of balance and tone training with the use of body weight. **Frequency**: 2 times/week **Intensity**: NR **Session duration:** NR **Primary exercise type**: Balance and functional **B. RT1:** Low-frequency, group-based supervised resistance training for upper and lower body with the use of resistance equipment. **Frequency**: 1 time/week **Intensity**: 2 sets of 8 RM **Session duration:** NR **Primary exercise type**: Resistance **C. RT2**: High-frequency, group-based supervised resistance training for upper and lower body with the use of resistance equipment. **Frequency**: 2 times/week **Intensity**: 2 sets of 8 RM **Session duration:** NR **Primary exercise type**: Resistance **Duration of the interventions (wks)**: 52 **Delivered by**: Certified fitness instructors	A vs B A vs C	1. Tibial volumetric cortical density (CovBMD) 2. Total area (ToA) midtibia 3. Tibial bone strength	12	Adjusted mean difference (95% CI) 1. Tibial CovBMD B – A 0.76 (-5.32 to 6.85) C – A -2.09 (-8.22 to 4.05) 2. Total area (ToA) midtibia B – A 0.10 (-2.72 to 2.92) C – A -0.49 (-3.34 to 2.35) 3. Tibial bone strength B – A 23.32 (-248.86 to 295.5) C – A -91.56 (-366.5 to 183.28)
^b^Blumenthal 1991 6/10 [44]	RCT 101/85	**Setting**: NR **Health status**: Healthy **A. Aerobic Training** ***n***= 33 (randomised) **B. Yoga and flexibility** ***n***= 34 (randomised) **C. Control:** Not relevant for this comparison. **Age**: (whole sample) 67 (min-max: 60-83) **Female**: NR	**A. Aerobic training:** Endurance training involving bicycle ergometry, brisk walking/jogging, and arm ergometry. **Frequency**: 3 times/week **Intensity**: 70% heart rate reserve **Session duration**: 60 min **Delivered by**: NR **Duration of intervention (wks):** 16 **Primary exercise type:** Endurance training **B. Yoga:** Supervised non-aerobic yoga programme. **Frequency**: at least 2 times/week **Intensity**: NR **Session duration**: 60 min **Delivered by:** NR **Duration of intervention (wks):** 16 **Primary exercise type**: Balance and function	A vs B	1. Distal radius BMD	14	1. Distal radius BMD: no between-group differences. Quantitative estimates not reported for between-group comparisons.
^d^Chan 2018 7 /10 [92]	RCT 110/54	**Setting**: Community; Taiwan **Health Status**: Increased risk for falls and fracture **A. Integrated care (IC)** ***n***= 55 (randomised); 31 (analysed) **Age**: 74.6 (7.4) **Female**: 69% **B. Lower extremity exercise (LEE)** ***n***= 55 (randomised); 23 (analysed) **Age**: 73.08 (6.57) **Female**: 69%	Both groups: 1-hour educational course related to osteoporosis, sarcopenia and 1-hour exercise intervention including warm up, brisk walking and gentle stretching. Subjects encouraged to conduct exercise at least 3 times per week. **A. Integrated care (IC):** Basic intervention, 15-minute warm-up exercise, 30-minute resistance exercise and 10-minute balance exercise **Frequency:** 1 time/week **Intensity**: Resistance training using rubber band and bottled water (0.6–1 L) as weight for upper and lower limbs. **Delivered by**: NR **Session duration**: 55 min **Duration of the intervention (wks)**: 12 **Primary exercise type:** Multiple (resistance plus balance and functional) **B. Lower extremity exercise (LEE):** Basic intervention and machine based lower extremity resistance exercise **Frequency:** 2 times/week **Intensity**: 60-80% of 1 RM **Delivered by**: NR **Session duration**: 30 min **Duration of the intervention (wks)**: 12 **Primary exercise type**: Resistance	A vs B	1. Lumbar spine BMD 2. Hip BMD	3	Change score (% change) 1. Lumbar spine BMD A. IC: 1.26% B. LEE: 2.08% 2. Hip BMD A. IC: -1.73% B. LEE: -0.88%
^b^Helge 2014 5/10 [50]	RCT 27/23	**Setting**: Community; Denmark **Health status**: Healthy **A. Football group** ***n***= 9 (randomised); 9 (analysed) **Age**: 68.0 (4.0) **B. Resistance training** ***n***= 9 (randomised); 8 (analysed) **Age**: 69.1 (3.1) **C. Control:** Not relevant for this comparison. **Female**: 0%	**A. Football group:** Supervised progressive football training **Frequency**: 1.7 (0.3) times/week (range: 1.2-2.2) **Intensity**: 82% of maximum heart rate (range 64 to 90%) **Session duration**: 45 to 60 min **Delivered by**: NR **Duration of the intervention (wks)**: 52 **Primary exercise type**: Balance and function (football) **B. Resistance training:** Progressive resistance training for core and upper and lower body **Frequency**: 1.9 (0.2) times/week (range: 1.4-2.2) **Intensity**: Started from 3 sets of 16-20 RM to 4 sets of 8 RM **Session duration**: 45 to 60 min **Delivered by**: NR **Duration of the intervention (wks)**: 52 **Primary exercise type**: Resistance (seated)	A vs B	1. Whole body BMD 2. Right femoral neck BMD 3. Left femoral neck BMD 4. Right femoral shaft BMD 5. Left femoral shaft BMD 6. Total right proximal femur 7. Total left proximal femur	12	Final score (mean ± SD) 1. Whole body BMD A. Football: 1.211 ± 0.036 B. Resistance: 1.225 ± 0.024 2. Right femoral neck BMD A. Football: 0.921 ± 0.034 B. Resistance: 1.000 ± 0.042 3. Left femoral neck BMD A. Football: 0.939 ± 0.034 B. Resistance: 1.006 ± 0.036 4. Right femoral shaft BMD A. Football: 1.156 ± 0.042 B. Resistance: 1.229 ± 0.056 5. Left femoral shaft BMD A. Football: 1.143 ± 0.043 B. Resistance: 1.229 ± 0.057 6. Total right proximal femur A. Football: 0.982 ± 0.031 B. Resistance: 1.066 ± 0.048 7. Total left proximal femur A. Football: 0.989 ± 0.031 B. Resistance: 1.069 ± 0.048
^b^Karinkanta 2007^c^ 7/10 [53]	RCT 149/144	**Setting**: Community; Finland **Health Status**: Healthy and excluded participants with osteoporosis **A. Balance-jumping training** ***n***= 37 (randomised); 35 (analysed) **Age**: 72.9 (2.3) **B. Resistance training** ***n***= 37 (randomised); 37 (analysed) **Age**: 72.7 (2.5) **C. Combined Balance-jumping and resistance training** ***n***= 38 (randomised); 36 (analysed) **Age**: 72.9 (2.2) **D. Control:** Not relevant for this comparison **Female**: 100%	**A. Balance-jumping training:** Balance training including static and dynamic balance exercise, agility training, impact exercises and changes of direction exercise. **Intensity**: NR **Primary exercise type**: Balance and function including bone loading (jumps) **B. Resistance training:** Tailored progressive resistance training programme for large muscle groups. **Intensity**: Initially 2 sets of 10-15 reps at intensity 50-60% of 1RM, progressed to 3 sets of 8-10 reps at 75-80% of 1RM. Rate of perceived exertion: above 18 out of 20 **Primary exercise type**: Resistance **C. Combined Balance-jumping and resistance training**: A combination of A & B on alternate weeks. **Primary exercise type**: Multiple (balance and function plus resistance) For all exercise groups: **Frequency**: 3 times/week **Session duration**: 50 min **Delivered by:** Exercise leaders **Duration of the intervention (wks)**: 52	A vs B A vs C B vs C	1. Femoral neck BMC 2. Distal tibia trabecular density (mg/cm^3^)	12	Final score (mean ± SD) 1. Femoral neck BMC A. Balance: 2.73 ± 0.40 B. Resistance: 2.71 ± 0.33 C. Combined: 2.65 ± 0.29 2. Distal tibia trabecular density (mg/cm^3^) A. Balance: 224 ± 34 B. Resistance: 219 ± 26 C. Combined: 215 ± 39
^eb^Karinkanta 2009^c^ 5/10 [98]	RCT 149/126	**Setting**: Community; Finland **Health Status**: healthy and excluded participants with osteoporosis **A. Balance jumping training group** ***n***= 37 (randomised); 33 (analysed) **Age**: 72.9 (2.3) **B. Resistance training group** ***n***= 37 (randomised); 34 (analysed) **Age**: 72.7 (2.5) **C. Combined resistance and balance jumping training group** ***n***= 38 (randomised); 32 (analysed) **Age**: 72.9 (2.2) **D. Non-training control group** ***n***= 27 (randomised); 27(analysed) **Age**: 72.0 (2.1) **Female:** 100%	**A. Balance-jumping training:** Balance training including static and dynamic balance exercise, agility training, impact exercises and changes of direction exercise. **Intensity**: NR **Primary exercise type**: Balance and function including bone loading (jumps) **B. Resistance training:** Tailored progressive resistance training programme for large muscle groups. **Intensity**: Initially 2 sets of 10-15 reps at intensity 50-60% of 1RM, progressed to 3 sets of 8-10 reps at 75-80% of 1RM. Rate of perceived exertion: above 18 out of 20 **Primary exercise type**: Resistance **C. Combined Balance-jumping and resistance training**: A combination of A & B on alternate weeks. **Primary exercise type**: Multiple (balance and function plus resistance) For all exercise groups: **Frequency**: 3 times/week **Session duration**: 50 min **Delivered by:** Exercise leaders **Duration of the intervention (wks)**: 52	A vs D B vs D C vs D	1. Femoral neck section moduls (Z) (mm^3^) 2. Tibia midshaft desnity-weighted polar section modulus (BSI) (mm^3^)	12	Mean change score (95% CI) reported on a graph Quantitative data was only reported between-group differences with control group as a reference. Additional results were reported in a graph.
^b^Kohrt 1997 3/10 [55]	Quasi-randomised trial 39/30	**Setting:** NR; United States **Health Status**: Healthy **A. Ground reaction forces training** ***n***= 14 (randomised); 12 (analysed) **Age**: 66.0 (1.0) **B. Joint reaction forces training** ***n***= 13 (randomised); 9 (analysed) **Age**: 65.0 (1.0) **C. Control:** Not relevant for this comparison **Female**: 100%	**A. Ground reaction forces training:** Individualised exercise training focusing on activities that involved ground-reaction forces, such as walking, jogging and/or stair climbing. **Frequency**: 3 to 5 times/week **Intensity**: 60-70% to 80-85% maximum heart rate **Session duration:** 30 to 45 min **Delivered by**: NR **Duration of the intervention (wks)**: 36 **Primary exercise type**: Multiple (balance and function plus endurance plus flexibility) **B. Joint reaction forces training:** Individualised exercise training including activities that involved joint-reaction forces, such as weightlifting and rowing. **Frequency**: 3 to 5 sessions/week **Intensity**: Weightlifting: 2-3 sets of 8-12 reps; Rowing: 60-70% to 80-85% of maximum heart rate **Session duration:** NR for the total session duration; however; rowing took 15 to 20 min **Delivered by**: NR **Duration of the intervention (wks)**: 36 **Primary exercise type**: Multiple (resistance plus endurance plus flexibility)	A vs B	1. Whole body BMD 2. Lumbar spine (L2-L4) BMD 3. Femoral neck BMD 4. Trochanter BMD 5. Ward’s BMD 6. Ultra distal wrist BMD 7. One-third distal wrist BMD	12	Quantitative estimates not reported (chance scores are provided in a graph) 1. Whole body BMD Positive effect towards “A” 2. Lumbar spine (L2-L4) BMD Positive effect towards “A” 3. Femoral neck BMD Positive effect towards “A” 4. Trochanter BMD Positive effect towards “A” 5. Ward’s BMD Positive effect towards “A” 6. Ultra distal wrist BMD Positive effect towards “B” 7. One-third distal wrist BMD Positive effect towards “B”
^b^Rikli 1990 1/10 [67]	Quasi-randomised trial 37/31	**Setting**: Local retirement community; United States **Health Status**: Healthy **A. General exercise** ***n***= 13 (randomised); 10 (analysed) **Age**: 72.2 (5.57) **B. General exercise + weight** ***n***= 13 (randomised); 10 (analysed) **Age**: 71.6 (5.66) **C. Control**: Not relevant for this comparison **Female**: 100%	**A. General exercise:** Group-based aerobic exercise training for large muscle groups. **Frequency**: 3 times/week **Intensity**: 60-70% maximum heart rate **Session duration**: 30 to 50 min **Delivered by**: NR **Duration of the intervention (wks)**: 40 **Primary exercise type:** Endurance **B. General exercise + weight:** Group-based aerobic exercise training plus upper body progressive resistance training. The resistance training was performed without supervision. **Frequency**: 3 times/week **Intensity**: 60-70% maximum heart rate for aerobic activities **Session duration**: 50 to 70 min **Delivered by**: Assistants **Duration of the intervention (wks)**: 40 **Primary exercise type**: Multiple (resistance plus endurance)	A vs B	1. Distal radius BMC/BW 2. Distal radius BMC	10	Change score (%) 1. Distal radius BMC/BW A. General exercise: 0.921 B. General exercise and weight: 1.734 2. Distal radius BMC A. General exercise: 1.023 B. General exercise and weight: 1.743 Statistical test not performed between the two intervention groups
Shen 2007 6/10 [69]	RCT 28/24	**Setting**: Local senior living campus; United States **Health Status**: Healthy **A. Tai chi** ***n***= 14 (randomised); 12 (analysed) **Age**: 78.8 (1.3) **Female**: 79% **B. Resistance exercise** ***n***= 14 (randomised); 12 (analysed) **Age**: 79.4 (2.2) **Female**: 71%	**A. Tai chi**: 24-form simplified Yang style Tai Chi. **Frequency:** 3 times/week **Intensity**: NR **Session duration**: 40 min **Delivered by**: Experienced Tai Chi instructor **Duration of the intervention (wks)**: 24 **Primary exercise type:** 3D (Tai Chi) **B. Resistance exercise:** Low-intensity resistance training for lower and upper extremities using equipment and dumbbells. **Frequency:** 3 times/week **Intensity**: 1 set of 10-12 reps at 50% of the 1RM **Session duration**: 40 min **Delivered by**: Certified fitness trainer **Duration of the intervention (wks)**: 24 **Primary exercise type**: Resistance	A vs B	1. Bone specific alkaline phosphatase (BAP), concentration change (%) 2. Pyridinoline (PYD), concentration change (%) 3. Parathyroid hormone (PTH), concentration change (%)	6	1. BAP No between-group difference (positive effect towards Tai chi) 2. PYD No between-group difference (positive effect towards Resistance training) 3. PTHP No between-group difference (positive effect towards Resistance training) Quantitative results not provided. Results reported in a graph
^*b*^*Woo 2007* 6/10 [75]	RCT 180/176	**Setting**: Community; Hong Kong **Heath status**: Healthy **A. Tai Chi** **n**=60 (randomised); 58 (randomised) **Age**: 68.2 years **B. Resistance training** ***n***= 60 (randomised); 59 (randomised) **Age**: 68.7 years **C. No Treatment**: Not relevant for this comparison **Female**: 50%	**A. Tai Chi:** 24-forms of Tai Chi using Yang style **Frequency:** 3 times/week **Intensity**: NR **Session duration**: NR **Delivered by**: NR **Duration of the intervention (wks)**: 52 **Primary exercise type**: 3D (Tai Chi) **B. Resistance training:** Resistance training with the use of medium strength TheraBand **Frequency:** 3 times/week **Intensity**: 30 times with medium strength TheraBand **Session duration**: NR **Delivered by**: NR **Duration of the intervention (wks)**: 52 **Primary exercise type**: Resistance	A vs B	1. Total hip BMD 2. Total spine BMD	12	Change score (mean % change ± SE) Men 1. Total hip BMD A. Tai Chi: -0.48 ± 0.37 B. Resistance: -1.20 ± 0.38 2. Total spine BMD A. Tai Chi: 1.35 ± 0.40 B. Resistance: 1.27 ± 0.42 Women 1. Total hip BMD A. Tai Chi: 0.07 ± 0.64 B. Resistance: 0.09 ± 0.62 2. Total spine BMD A. Tai Chi: 0.10 ± 0.50 B. Resistance: 1.98 ± 0.48

*BMC* bone mineral content (g), *BMD* bone mineral density (g/cm^2^), *BMI* body mass index (kg/m^2^), *BW* bone width, *NR* not reported, *RCT* randomised controlled trial. When data was available for more than one time-point, we extracted the post-intervention and follow-up data. Mean estimates were extracted in the following hierarchical order: mean difference, change score and final score

^a^Exercise is a physical activity that is planned, structured and repetitive and aims to improve or maintain physical fitness. There is a wide range of possible types of exercise, and exercise programmes often include one or more types of exercise. We categorised exercise based on a modification of the Prevention of Falls Network Europe (ProFaNE) taxonomy that classifies exercise type as: i) gait, balance, and functional training; ii) strength/ resistance (including power); iii) flexibility; iv) three- dimensional (3D) exercise (e.g., Tai Chi, Qigong, dance); v) general physical activity; vi) endurance; and vii) other kind of exercises. The taxonomy allows for more than one type of exercise to be delivered within a programme. We also considered whether the exercise explicitly included bone loading eg hopping or heel drops

^b^Indicates studies also included in the exercise vs control comparison (Table 1), but only the results for exercise comparisons are presented here

^c^indicates articles reporting results from the same study

^d^indicates studies that were found in the expanded search for individual studies conducted in March 2020 in PubMed

^e^indicates studies that were found in the updated search for systematic reviews conducted in July 2020 in PubMed, Embase, CINAHL, SPORTDiscus

^¥^ indicates statistically significant between-group differences at *p* < 0.05.

**Table 3:** Description of included studies investigating the association between different doses of physical activity on osteoporosis prevention

Reference PEDro score	Study design Allocated/ Analysed	Participants (n, age mean (SD), % women, setting, health status)	Intervention Primary exercise type according to ProFANE^**a**^	Relevant comparison	Outcomes	Follow up (mo)	Results
^b^Ashe 2013 [77]	RCT 155/147	**Setting**: Community; Canada **Health status**: Healthy **A. Balance and tone (BT)** Not relevant for this comparison **B. Once a week resistance training (RT1)** ***n***= 54 (randomised); 47 (analysed) **Age**: 69.4 (3.0) **C. Twice a week resistance training (RT2)** ***n***= 52 (randomised); 46 (analysed) **Age**: 69.2 (3.0) **Female**: 100%	**B. RT1:** Low-frequency, group-based supervised resistance training for upper and lower body with the use of resistance equipment. **Frequency**: 1 time/week **Intensity**: 2 sets of 8 RM **Session duration:** NR **Primary exercise type**: Resistance **C. RT2**: High-frequency, group-based supervised resistance training for upper and lower body with the use of resistance equipment. **Frequency**: 2 times/week **Intensity**: 2 sets of 8 RM **Primary exercise type**: Resistance **Duration of the interventions (wks)**: 52 **Delivered by**: Certified fitness instructors	B vs C	1. Tibial volumetric cortical density (CovBMD) 2. Total area (ToA) midtibia 3. Tibial bone strength	12	Final score (mean ± SD) 1. Tibial CovBMD B. -1.81 ± -0.17 C. -4.67 ± -0.45 2. Total area (ToA) midtibia B. 0.86 ± 0.21 C. 0.93 ± 0.22 3. Tibial bone strength B. 124.83 ± 0.64 C. 9.94 ± 0.05
^c^Bemben 2011 4/10 [91]	RCT 160/124	**Setting**: Community; United States **Health Status**: Healthy **Age**: Men 65.2 (0.5); Female 63.8 (0.4) **Female**: 64% **A. 2 days/week high intensity (2HI) group** ***n***=39 (randomised); 31 (analysed) **B. 2 days/week low intensity (2LI) group** **n**=41 (randomised); 34 (analysed) **C. 3 days/week high intensity (3HI) group** **n**=34 (randomised); 24 (analysed) **D. 3 days/week low intensity (3LI)** ***n***= 46 (randomised); 35 (analysed)	Training included five upper body and seven lower body exercise **A. 2HI:** **Frequency**: 2 times/week **Intensity**: 80% of 1RM, 3 sets of 8 reps **Session duration:** 60 min **Delivered by**: NR **Duration of the intervention (wks)**: 40 **Primary exercise type**: Resistance **B**. **2LI:** **Frequency**: 2 times/week **Intensity**: 40% of 1RM, 3 sets of 16 reps **Session duration:** 60 min **Delivered by**: NR **Duration of the intervention (wks)**: 40 **Primary exercise type**: Resistance **C**. **3HI:** **Frequency**: 3 times/week **Intensity**: 80% of 1RM, 3 sets of 8 reps **Session duration:** 60 min **Delivered by**: NR **Duration of the intervention (wks)**: 40 **Primary exercise type**: Resistance **D**. **3LI:** **Frequency**: 3 times/week **Intensity**: 40% of 1RM, 3 sets of 16 reps **Session duration:** 60 min **Delivered by**: NR **Duration of the intervention (wks)**: 40 **Primary exercise type**: Resistance	A vs B A vs C C vs D B vs D	1. Lumbar spine (L2-4) BMD 2. Femoral neck BMD 3. Trochanter BMD 4. Total hip BMD 5. Total body BMD	10	Final score (mean ± SD) 1. Lumbar spine BMD A. 2HI: 1.155 ± 0.034 B. 2LI: 1.195 ± 0.034 C. 3HI: 1.190 ± 0.034 D. 3LI: 1.190 ± 0.031 2. Femoral neck BMD A. 2HI: 0.902 ± 0.020 B. 2LI: 0.904 ± 0.019 C. 3HI: 0.889 ± 0.021 D. 3LI: 0.932 ± 0.027 3. Trochanter BMD A. 2HI: 0.792 ± 0.025 B. 2LI: 0.781 ± 0.019 C. 3HI: 0.800 ± 0.025 D. 3LI: 0.811 ± 0.031 4. Total hip BMD A. 2HI: 0.949 ± 0.022 B. 2LI: 0.943 ± 0.019 C. 3HI: 0.956 ± 0.025 D. 3LI: 0.984 ± 0.031 5. Total body BMD A. 2HI: 1.172 ± 0.014 B. 2LI: 1.175 ± 0.015 C. 3HI: 1.199 ± 0.020 D. 3LI: 1.177 ± 0.017^**¥**^
Kemmler 2010 6/10 [54]	RCT 246/227	**Setting**: Community; Germany **Health status**: Healthy **A. Multi-component exercise training** ***n***= 123 (randomised); 115 (analysed) **Age**: 68.9 (3.9) **B. Low intensity multicomponent programme** ***n***= 123 (randomised); 112 (analysed) **Age**: 69.2 (4.1) **Female**: 100%	**A.** Two 60-minute supervised group sessions: warm-up/ aerobic dance (20 min), balance training (5 min); functional gymnastics, isometric strength training with 1-3 sets of isometric floor exercises for trunk flexors and extensors hip flexors and extensors and leg abductors and adductors; upper body exercises. Two home training session that includes strength and flexibility training. **Frequency**: 4 sessions/week **Intensity**: Aerobic dance: 70%-85% of maximum heart rate; Upper body exercise: 10-15 reps x 2-3sets; Home training session: 1-2 sets of 6-8 isometric exercise and 10-15 reps x 2 sets of belt exercises **Session duration:** 60 min/group class & 20 min/home training session **Delivered by**: Certified trainer **Duration of the intervention (wks)**: 72 **Primary exercise type**: Multiple (balance and function plus resistance) **B**. Low intensity multicomponent programme including walking, muscular relaxation, endurance and strength training **Frequency:** 1 session/week **Intensity**: Walking at 50-60 % maximum heart rate Endurance and strength training: low to moderate intensity **Session duration:** 60 min **Delivered by**: Certified trainer **Duration of the intervention (wks)**: 72 [every 10 weeks of training was followed by 10 weeks of rest] **Primary exercise type**: Multiple (balance and function plus endurance)	A vs B	1. Lumbar spine BMD 2. Femoral neck BMD	18	Mean difference (95% CI) 1. Lumbar spine BMD: 0.014 (0.006 to 0.021) 2. Femoral neck BMD: 0.015 (0.008 to 0.021)^**¥**^
^b^Pruitt 1995 4/10 [64]	RCT 40/26	**Setting**: Community; America **Healthy status**: Healthy **A. High intensity resistance training** ***n***= 15 (randomised); 8 (analysed) **Age**: 67.0 (0.5) **B. Low intensity resistance training** ***n***= 13 (randomised); 7 (analysed) **Age**: 67.6 (1.4) **C. Control:** not relevant for this comparison **Female**: 100%	**A and B**. Supervised exercise session comprising bench press, lateral pull down, military press, biceps curl, knee extension, knee flexion, hip abduction and adduction, leg press, back extension. **A**. **Intensity:** High 14 reps x 1 set at 40% 1RM for warm up; 7 reps x 2 sets at 80% 1RM **B. Intensity:** Low 14 reps x 3 sets at 40% 1RM For both A and B: **Frequency**: 3 times/week **Session duration**: 50 -55min/lifting time **Delivered by**: NR **Duration of the intervention (wks)**: 52	A vs B	1. Total hip BMD 2. Femoral neck BMD 3. Ward’s triangle BMD 4. Lumbar spine (L2-L4) BMD	12	Change score (mean ± SD) 1. Total hip BMD A. High intensity: 0.005 ± 0.014 B. Low intensity: 0.008 ± 0.012 2. Femoral neck BMD A. High intensity: -0.002 ± 0.154 B. Low intensity: 0.025 ± 0.008 3. Ward’s triangle BMD A. High intensity: 0.018 ± 0.032 B. Low intensity: 0.022 ± 0.045 4. Lumbar spine (L2-L4) BMD A. High intensity: 0.007 ± 0.018 B. Low intensity: 0.005 ± 0.027
^d^Taaffe 1996 4/10 [101]	RCT 36/21	**Setting**: Community; United States **Health status**: Healthy **A. High intensity resistance training group** ***n***= 12 (randomised); 7 (analysed) **Age**: 67.0 (0.2) **B. Low intensity resistance training group** ***n***= 13 (randomised); 7 (analysed) **Age**: 67.6 (0.5) **C. Control** ***n***= 11 (randomised); 7 (analysed) **Age**: 69.6 (1.3) **Female**: 100%	Supervised exercise training targeted thigh muscle strength including leg press, knee extension and knee flexion. Exercise sessions were bracketed by warm up and cool-down periods **A. Intensity**: 1 set of 14 reps at an intensity of 40% 1RM and 2 sets of 7 reps at an intensity of 80% of 1RM **B. Intensity:** 3 sets of 14 reps at an intensity of 40% of 1RM For both groups **Frequency**: 3 times/week **Session duration**: NR **Delivered by:** NR **Duration of the intervention (wks)**: 52 **Primary exercise type**: Resistance	A vs B A vs C B vs C	1. Middle third of the femur BMD 2. Thigh BMD	12	% Change score (mean ± SEM) 1. Middle third of the femur BMD^**¥**^ A. High intensity: 1.0± 1.0 B. Low inteisty: -2.2 ± 0.5 C. Control: -1.8 ± 0.6 2. NR
^b^Taaffe 1999 5/10 [72]	RCT 53/46	**Setting**: Community; United States **Health status**: Healthy **A. High-intensity resistance training (1 day per week)** ***n***= 14 (randomised); 11 (analysed) **Age**: 68.5 (3.6) **Female**: 36% **B. High-intensity resistance training (2 days per week)** ***n***= 14 (randomised); 12 (analysed) **Age**: 69.4 (3.0) **Female**: 29% **C. High-intensity resistance training (3 days per week**) ***n***= 11 (randomised & analysed) **Age**: 71.0 (4.1) **Female**: 36% **D. Control**: not relevant for this comparison	Training includes the whole body (bench press, military press, latissimus pull-down, biceps curl, and leg press) All trainings were started with a warm up that included stretching and one set each of bench press and leg press (40% of 1-RM, 10 repetitions) and concluded with a cool-down period of stretching. Intensity: started at 60% of the 1RM and gradually increase in intensity **A. Frequency**: 1 time/week **B. Frequency**: 2 times/week **C. Frequency**: 3 times/week **Intensity**: 8 reps x 3 sets at 80% of 1 RM **Session duration**: NR **Delivered by**: NR **Duration of the intervention (wks)**: 24	A vs B A vs C B vs C	1. Lumbar spine (L2-L4) BMD 2. Total hip BMD 3. Midradius BMD 4. Total body BMC	6	Final score (mean ± SD) 1. Lumbar spine (L2-L4) BMD A. Resistance 1x/week: 1.025 ± 0.006 B. Resistance 2x/week: 1.033 ± 0.006 C. Resistance 3x/week: 1.032 ± 0.007 2. Total hip BMD A. Resistance 1x/week: 0.865 ± 0.010 B. Resistance 2x/week: 0.866 ± 0.006 C. Resistance 3x/week: 0.864 ± 0.010 3. Midradius BMD A. Resistance 1x/week: 0.605 ± 0.003 B. Resistance 2x/week: 0.604 ± 0.003 C. Resistance 3x/week: 0.608 ± 0.003 4. Total body BMC A. Resistance 1x/week: 2552 ± 15 B. Resistance 2x/week: 2530 ± 14 C. Resistance 3x/week: 2525 ± 14

BMC: bone mineral content (g); BMD: bone mineral density (g/cm^2^); BMI: body mass index (kg/m^2^); NR: not reported; RCT: randomised controlled trial. When data was available for more than one time-point, we extracted the post-intervention data. Mean estimates were extracted in the following hierarchical order: mean difference, change score and final score

^a^Exercise is a physical activity that is planned, structured and repetitive and aims to improve or maintain physical fitness. There is a wide range of possible types of exercise, and exercise programmes often include one or more types of exercise. We categorised exercise based on a modification of the Prevention of Falls Network Europe (ProFaNE) taxonomy that classifies exercise type as: i) gait, balance, and functional training; ii) strength/ resistance (including power); iii) flexibility; iv) three- dimensional (3D) exercise (e.g., Tai Chi, Qigong, dance); v) general physical activity; vi) endurance; and vii) other kind of exercises. The taxonomy allows for more than one type of exercise to be delivered within a programme. We also considered whether the exercise explicitly included bone loading eg hopping or heel drops

^b^Indicate studies also included in the exercise vs control comparison (Table 1) or in the one or more forms of physical activity comparison (Table 2), but only the results for different doses of exercise are presented here

^c^indicates studies that were found in the expanded search for individual studies conducted in March 2020 in PubMed

^d^indicates studies that were found in the updated search for systematic reviews conducted in July 2020 in PubMed, Embase, CINAHL, SPORTDiscus

^**¥**^indicates statistically significant between-group difference at *p* < 0.05

**Table 4:** Description of included studies investigating the association between physical activity and osteoporosis employing an observational design

Reference Overall Risk of Bias	Study design Included / Analysed	Participants (n, age mean (SD), % women, setting, health status)	Exposure	Outcomes	Results
^a^Foley 2010 (Tasmania Older Adult Cohort study) Overall risk of bias: High [81]	Prospective cohort study (2.6 years follow-up) 875	**Setting:** Community, Australia **Health status:** Healthy ***n***= 875 (included) **Age:** 62.7 (7.3) (included) [NB: quartile 1, 2 and 3 are not of interest in this study as mid-point age <65 years] **Female:** 49% **Mid-point of age quartile 4:** Age: 74.8	Ambulatory activity (steps per day) was assessed using pedometer for 1 week at both baseline and follow-up and participants were divided in quartiles **Classification**: total physical activity	1. Lumbar spine areal BMD 2. Hip areal BMD	Adjusted point estimates (95% CIs) 1. Lumbar spine areal BMD Not reported for sample >65 years 2. Hip areal BMD at follow-up Age quartile 4, Female Q1. 0.434 (0.372 to 0.497) Q2. 0.441 (0.378 to 0.503) Q3. 0.446 (0.383 to 0.509) Q4. 0.466 (0.401 to 0.532) Age, quartile 4, Male Q1. 0.554 (0.490 to 0.618) Q2. 0.566 (0.501 to 0.631) Q3. 0.572 (0.507 to 0.637) Q4. 0.584 (0.518 to 0.651)
^a^Muir 2013 (Canadian multicentre osteoporosis study) Overall risk of bias: Low [84]	Retrospective (previous 12 months)	**Setting:** Community; Canada **Health status:** Mixed (included participants with diagnosis of osteoporosis) ***n***= 1169 **Age:** 79.84 (4.43) **Female:** 100%	Physical activity was quantified based on the level of activity (moderate or strenuous or vigorous) and the reported frequency and duration of said activity over the course of the previous 12 months. **Classification**: total physical activity	1. Lumbar Spine (L1-4) BMD 2. Femoral neck BMD 3. Total hip BMD 4. Ward’s triangle BMD 5. Trochanter BMD	Multiple regression analysis of the relative effects of moderate activity on BMD. Coefficient (95% CI) 1. Lumbar Spine (L1-4) BMD -0.006 (-0.013 to 0.000) 2. Femoral neck BMD 0.004 (0.000 to 0.008)^¥^ 3. Total hip BMD 0.006 (0.001 to 0.011)^¥^ 4. Ward’s triangle BMD 0.004 (-0.001 to 0.009) 5. Trochanter BMD 0.005 (0.006 to 0.074)^¥^
^a^Nakamura 2012 (Muramatsu Study) Overall risk of bias: High [85]	Cohort (6 years follow-up) 774/382	**Setting:** Community; Japan **Health status:** Healthy ***n***= 382 **Age:** 73.3 (3.7) **Female:** 100%	Physical activity was assessed via questionnaire based on whether participants regularly engaged in light or moderate physical activity (yes/no) activities: **A. Light physical activity** (includes croquet, taking walks and traditional Japanese dancing): yes or no **B. Moderate physical activity** (includes farm work and gardening): yes or no **Classification**: total physical activity (light and moderate)	1. Forearm BMD	p-value association between baseline physical activity levels BMD changes 1. Forearm BMD A. Light: *p*=0.5122 or B. Moderate: *p*=0.0711 [Quantitative estimates not reported]
^a^Rodriguez-Gomez 2019 (Toledo Study for Healthy Aging) Overall risk of bias: High [86]	Cohort (4 years follow-up) 227/192	**Setting:** Community; United States **Health status:** Healthy ***n***= 192 **Age:** 80.5 (4.3) **Female: 52.6%**	Physical activity was assessed by accelerometry during waking hours for seven consecutive days, except while bathing or swimming activities **A. Sedentary behaviours** - mean % of waking hours: 55 (baseline); 59 (follow-up) **B. Light physical activity** - mean % of waking hours: 41.9 (baseline); 38.8 (follow-up) **C. Moderate to vigorous physical activity** – mean % of waking hours: 3.1 (baseline); 2.2 (follow-up) **Classification**: total physical activity	1. Whole body BMC 2. Pelvic BMC 3. Arms (mean) BMC 4. Legs (mean) BMC 5. Lumbar spine (L1-4) BMC 6. Femoral regions BMC (proximal femur – mean, femoral neck, trochanter, ward’s triangle) 7. Whole body BMD 8. Pelvic BMD 9. Arms (mean) BMD10. Legs (mean) BMD 11. Lumbar spine (L1-4) BMD 12. Femoral regions BMD (proximal femur – mean, femoral neck, trochanter, ward’s triangle)	NB: Only significant findings were reported here] Multiple regression coefficient (y) of change in the composition of movement behaviours and changes in BMC or BMD: 4. Legs (mean) BMC A. NS B. NS C. y=1.767, *p*= 0.04^¥^ 5. Lumbar spine (L1-4) BMC A. NS B. NS C. y=0.050, *p*= 0.03^¥^ 10. Legs (mean) BMD A. NS B. NS C. y=0.005, *p*= 0.04^¥^
^a^Shephard 2017 (Nakanojo Study) Overall risk of bias: Low [87]	Cohort (5 years follow-up) 615/496	**Setting:** Community**;** Japan **Health status:** Healthy Men **Age:** 71.2 (3.9) ***n***= 212 Women **Age:** 71.3 (4.2) ***n***= 284 **Female**: 57.3%	Physical activity was measured using pedometer for 5 years and analysed as daily step count and the daily duration of exercise at an intensity >3 METs **A. Physical activity level Quartile 1** Step count (steps/day) at baseline: 3888 (1117) [men]; 3824 (1298) [women] Duration of activity > 3 METs (min/day): 4.0 (1.8) [men]; 4.0 (2.4) [women] **B. Physical activity level Quartile 2** Step count (steps/day) at baseline: 5994 (943) [men]; 5931 (924) [women] Duration of activity > 3 METs (min/day): 10.9 (2.4) [men]; 10.1 (2.3) [women] **C. Physical activity level Quartile 3** Step count (steps/day) at baseline: 7521 (833) [men]; 7626 (691) [women] Duration of activity > 3 METs (min/day): 19.3 (2.9) [men]; 18.4 (2.6) [women] **D. Physical activity level Quartile 4** Step count (steps/day) at baseline: 10892 (1433) [men]; 10199 (1398) [women] Duration of activity > 3 METs (min/day): 31.8 (5.6) [men]; 30.3 (4.3) [women] **Classification**: total physical activity	1. Osteosonic Index (OSI) from the ultrasonic measurement of the calcaneus (Fracture threshold – yes/no)	Multivariate Cox proportional hazard ratio (risk of the OSI falling below the fracture threshold) and 95% CI Step count (steps/day) Men Q1. 2.63 (1.35 to 4.41)^¥^ Q2. 1.75 (1.03 to 3.95)^¥^ Q3. 1.01 (0.55 to 3.37) Q4. 1 Women Q1. 3.33 (2.10 to 5.21)^¥^ Q2. 2.51 (1.25 to 4.03)^¥^ Q3. 1.12 (0.47 to 2.16) Q4. 1 Duration of activity >3 METs (min/day) Men Q1. 2.77 (1.46 to 5.59)^¥^ Q2. 1.91 (1.02 to 3.99)^¥^ Q3. 1.00 (0.48 to 2.27) Q4. 1 Women Q1. 3.94 (2.35 to 6.73)^¥^ Q2. 1.87 (1.00 to 3.60)^¥^ Q3. 0.99 (0.40 to 2.06) Q4. 1
^a^Svejme 2014 Overall risk of bias: Low [88]	Cohort (25 years follow-up)	**Setting:** Community; Sweden **Health status:** Healthy **A. Active women** ***n***= 91 **B. Inactive women** ***n***= 21 **Age:** women recruited at age 48 and followed up for 25 years **Female:** 100%	Physical activity measured using questionnaires at four defined time periods: at menopause, 5 and 10 years after menopause, and at age 72. **A. Active women** (>30 min/day) – mean (95% CI) number of hours of physical activity per week: Baseline: 9.0 (7.8 to 10.2) Average post-menopausal physical activity: 8.7 (7.6 to 9.8) Physical activity at age 72: 8.2 (6.9 to 9.4) **B. Inactive women** (<30 min/day) – mean (95% CI) number of hours of physical activity per week: Baseline: 3.0 (1.6 to 4.4) Average post-menopausal physical activity: 2.0 (1.4 to 2.5) Physical activity at age 72: 1.2 (0.5 to 1.9) **Classification**: total physical activity	1. Forearm BMC (mg/cm) 2. Forearm bone mineral apparent density (mg/cm^3^)	Mean (95% CI) average annual changes 1. Forearm bone mineral content (mg/cm): A. -1.2 (-1.3 to -1.1) B. -1.6 (-1.9 to -1.3) Mean differences 0.4 (0.1 to 0.6)^¥^ 2. Forearm bone mineral apparent density A. -1.8 (-1.9 to -1.7) B. -2.0 (-2.2 to -1.7)
^a^Bleicher 2013 (CHAMP study) Overall risk of bias: Low [80]	Longitudinal cohort study (2 years follow-up) 1,705/1,122	**Setting:** Community; Australia **Health status:** Healthy ***n***= 1,122 **Age:** 76.2 (5.1); range 70-97 **Female:** 0%	**A.** Walking for daily exercise was self-reported and measured in kilometres per day **A**. Walk daily > 0 to ≤ 1km **B**. Walk daily > 1 to ≤ 2 km **C**. Walk daily > 2 to ≤ 4 km **D**. Walk daily > 4 km **Classification**: planned physical activity (exercise) **B.** Physical Activity Scale for the Elderly (units) **Classification:** total physical activity	1. Total hip BMD 2. Total hip BMC	Multiple regression coefficient (95% CI) Reference: no walking Leisure-time walking 1. Total hip BMD A. 0.09 (-0.18 to 0.36), *p*=0.5 B. 0.18 (-0.06 to 0.41), *p*=0.1 C. 0.29 (0.06 to 0.52), *p*=0.01^¥^ D. 0.19 (-0.1 to 0.49), *p*=0.2 2. Hip BMC: NR Age adjusted annualised percentage change in total hip BMD per unit change General physical activity 1. Total hip BMD -0.01 (-0.09 to 0.07) 2. Hip BMC: NR
Greendale 1995 (Rancho Bernardo study) Overall risk of bias: Low [49]	Retrospective study 1,703	**Setting**: Community; United States **Health status**: Healthy ***n***= 1,703 **Age**: 73 **Female**: 60%	Lifetime leisure physical activity, calculated based on leisure time physical activity (collected retrospectively via questionnaire) for the past year, age 30 years and age 50 years Exercise level: classified by the highest level of exercise performed for at least 15 minutes per session at least three times per week. Participants were divided into levels of physical activity according to the tertiles **A.** Low **B.** Medium **C**. High **Classification:** planned physical activity (exercise)	1. Total hip BMD 2. Intertrochanter BMD 3. Femoral neck BMD 4. Greater trochanter BMD 5. Lumbar spine (L1-4) BMD 6. Distal radius BMD 7. Midshaft radius BMD	Adjusted mean (p-value for comparison A vs C) 1. Total hip BMD (*p*=0.002)^**¥**^ A. Low: 0.8241 B. Medium: 0.8367 C. High: 0.8507 2. Intertrochanter BMD (*p*=0.007)^**¥**^ A. Low: 0.9631 B. Medium: 0.9769 C. High: 0.9908 3. Femoral neck BMD (*p*=0.003)^**¥**^ A. Low: 0.6597 B. Medium: 0 6716 C. High: 0.6819 4. Greater trochanter BMD (p = 0.0001)^**¥**^ A. Low: 0.5969 B. Medium: 0.6093 C. High: 0.6248 5. Lumbar spine (L1-4) BMD A. Low: 0.9324 B. Medium: 0.9612 C. High: 0.9479 6. Distal radius BMD: NR 7. Midshaft radius BMD: NR
^a^Gudmundsdottir 2010 Overall risk of bias: High [82]	Cohort (4 years follow-up) 162	**Setting:** Community**;** Iceland **Health status:** Healthy **A. Physical activity performed ≤ 3 per week** ***n***= 41 (analysed) **B. Physical activity performed > 3 per week** ***n***= 111 (analysed) **Age**: 75 **Female:** 100%	Physical activity was calculated based on number of leisure time walks per week and number of other exercise session per week (self-reported questionnaire) Results were presented according to number of times of physical activity performed per week **A. ≤ 3 per week** **B. > 3 per week** **Classification**: planned physical activity (exercise)	1. Femoral neck BMD 2. Total trochanter BMD 3. Total hip BMD	Change score; mean % change (SE) 1. Femoral neck A. -1.3 (1.1) B. -0.2 (0.9) β: NR 2. Total trochanter BMD A. -1.5 (0.8) B. -1.2 (0.7) β= 0.22, non-significant p value 3.Total hip BMD Mean (SE) A. -1.4 (0.8) B. -1.1 (0.7) β= 0.19, non-significant p value
Huddleston 1980 Overall risk of bias: High [51]	Retrospective observational study 35/35	**Setting**: Community; United States **Health status**: Healthy tennis athletes ***n***= 35 **Age**: range 70-79 **Female**: 0%	Lifetime tennis exposure in athletes with tennis experience ranging from 25 to 72 years Results were presented for: **A.** Playing arm **B.** Non-playing arm **C.** Comparison with data for a “normal male population” **Classification:** planned physical activity (sport – tennis)	1. Radius midshaft BMC	1. Radius BMC 4% to 33% greater for the playing arms as compared with the nonplaying arms The mean difference between the playing arm and nonplaying arm: 13% Reference data suggest difference between dominant and nondominant BMC values of 6% to 9%
^a^Kemmler (2016) Bone (Erlangen Fitness and Osteoporosis Prevention Study) Overall risk of bias: High [83, 93]	Retrospective secondary analysis of the intervention group of a quasi-randomised trial (16 years follow-up)	**Setting**: Community; Germany **Health status**: Osteopenia **A. Exercise group:** ***n***= 55 **Age**: 55.1 (3.4) **Female**: 100%	Exercise group: Supervised group class (aerobic dance exercise, jumping and resistance exercise) + Home training (rope skipping, isometric and dynamic resistance exercise and stretching/ flexibility exercise) five months after study started; 49 to 50 weeks/year throughout the 16 years Exercise frequency (ExFreq): session/week/16 years **Classification**: planned physical activity (exercise)	1. Lumbar spine BMD 2. Total hip BMD	Linear mixed-effect regression analysis. Marginal effect (95% CI) 1. Lumbar spine BMD: 0.035 (0.024 to 0.045) 2. Total hip BMD: 0.015 (0.005 to 0.026) Minimum effective dose of exercise (training sessions/week) 1. Lumbar spine: 2.11 (2.06 to 2.12) 2. Total hip BMD: 2.22 (2.00 to 2.78)
Rikkonen 2010 (Kuopio Osteoporosis Risk Factor and Prevention study) Overall risk of bias: Low [66]	Cohort (15 years follow-up) 8560	**Setting**: Community; Finland **Health status:** Healthy ***n***= 8560 (analysed) **A. Physical activity quartile I** **Age**: 52.1 (2.9) **B. Physical activity quartile II** **Age**: 52.0 (2.9) **C. Physical activity quartile III** **Age**: 52.2 (2.9) **D. Physical activity quartile IV** **Age**: 52.3 (2.8) **Female**: 100%	Leisure-time physical activity (self-reported) collected at 5 years intervals **A.** 15-year average PA, hours/week: 0.35 (0.35) **B**. 15-year average PA, hours/week: 1.7 (0.39) **C**. 15-year average PA, hours/week: 3.2 (0.54) **D**. 15-year average PA, hours/week: 7.0 (2.9) **Classification**: leisure-time physical activity (exercise, transportation, sport)	1. Femoral neck BMD 2. Trochanter BMD 3. Ward’s triangle BMD 4. Lumbar spine (L2-4) BMD	Beta ± SE (quartile IV vs inactive) 1. Femoral neck BMD 1.752 ± 0.493 2. Trochanter BMD 1.783 ± 0.581 3. Ward’s triangle BMD 2.412 ± 0.723 4. Lumbar spine (L2-4) BMD 0.040 ± 0.649 All results were significant (except for lumbar spine) and suggest a positive effect of physical activity on BMD.

*BMC* bone mineral content (g unless specified), *BMD* bone mineral density (g/cm2). Where studies reported effect estimates with differing degrees of adjustment for confounders in different models, we used the estimate from the most adjusted model

^a^indicate studies that were found in the updated search

^**¥**^indicates statistically significant between-group difference at *p* < 0.05.

The included trials comprised a wide range of physical activity and exercise modalities. Following the ProFaNE taxonomy, most studies (*n* = 19) investigated more than one category of exercise (classified as multiple); 11 studies investigated balance and functional exercises, 12 resistance; five endurance; nine investigated a combination of balance and functional exercise or resistance with bone loading; and one 3D exercises (Tai Chi).

### Participant characteristics

Most included studies recruited from the general older population. Studies in which all participants had already been diagnosed with osteoporosis were excluded. Four studies excluded participants with osteoporosis at baseline [53, 93, 95, 96]. Three studies included participants on the basis of having some level of frailty [46, 73, 74]; five articles reporting results from two studies included only participants with osteopenia [77, 81, 91, 92, 94]; two studies included only obese participants [87, 88]; two studies investigated prostate cancer survivors without osteoporosis [93, 95]; one study included participants who had had surgical repair of a hip fracture no more than 16 weeks prior to study entry [43]; and one study included participants with increased risk for falls and fracture [90]. One study investigated lifelong tennis athletes. Twenty-eight studies included only women whereas six investigated only men. Five studies (reported in 8 articles) included participants who were younger than 65 years at study entry, but met the age criteria at follow-up [66, 77, 81, 86, 91, 92, 94, 98].

### Outcomes

The included studies reported results for a range of different outcomes (*n* = 32), and the most common ones were measures of BMD and BMC. We performed an overall assessment of the evidence according to the study’s main outcome. If the study did not specify a main outcome, we selected the outcome we considered to be most relevant to the intervention (e.g., whole body for exercises involving the whole body). We selected lumbar spine in preference to hip when both were presented, and the exercise was primarily undertaken in a standing position. Where exercises were mostly performed in non-standing positions (e.g., seated, supine) and targeted the lower limb, hip measures were preferred. For studies that reported multiple hip measures, preference was given to total hip measures, if available. Preference was given to BMD when compared to other measures, such as BMC. We undertook two additional assessments according to the two most commonly reported outcomes across the included studies, which were measures of femoral neck BMD and lumbar spine BMD.

### Methodological quality of studies

The overall quality of included trials was moderate (median 5, range 1 to 7). The PEDro total scores are reported for all relevant studies in Tables 1, 2, and 3 and the scores for each item are reported in Additional file 4, Table 1. The overall risk of bias of longitudinal studies using the modified QUIPS tool is reported in Table 4. Six longitudinal studies had low risk of bias (Additional file 4, Table 2). The most common sources of bias were related to exposure measurement, study attrition and study confounding.

### Association between physical activity and osteoporosis prevention

**Fig. 2:**
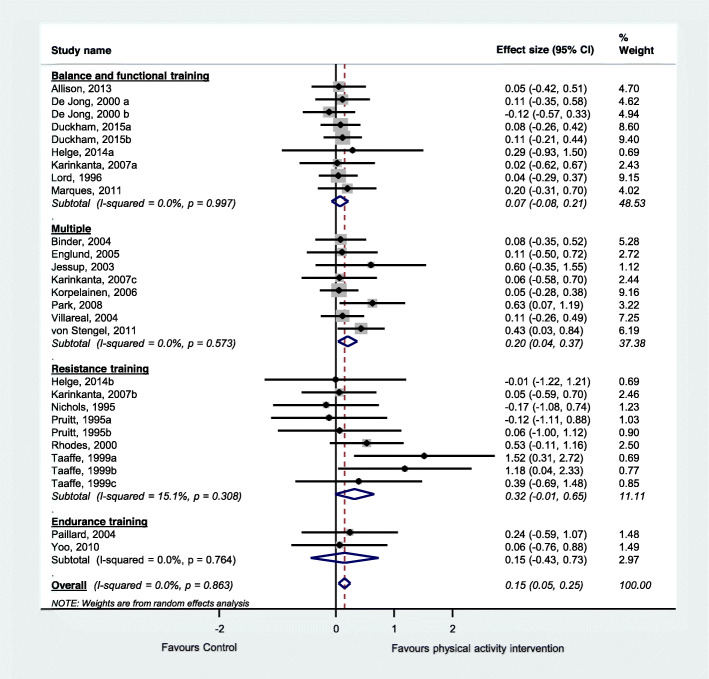
Effect size (95% confidence interval) of physical activity interventions on the main outcome by pooling data from 20 studies comparing physical activity versus control using random-effects meta-analysis (*n* = 1560)

A total of 40 articles reporting on 37 studies (30 randomised and 7 quasi-randomised trials) investigated physical activity interventions compared with a control group (Table 1). Overall the sample size for the trials was small (median: 50, range: 16 to 283) and the median follow-up length was 12 months (range 3 to 144). Meta-analysis revealed a significant but relatively small overall effect of exercise when the results of the main outcome from each study were pooled (standardised effect size 0.15, 95% CI 0.05 to 0.25, 20 trials, Fig. 2). The quality of evidence was moderate as per GRADE system, downgraded for study limitations, meaning that the true effect is likely to be close to the estimated results (Table 5 and Additional file 5, Supplementary Table A). The overall results suggest that physical activity interventions probably improve bone health and prevent osteoporosis in older adults.

**Table 5:** Summary of findings and quality of evidence assessment for physical activity interventions vs control

Outcome	Summary of findings		Quality of evidence assessment (GRADE)				
	Effect size^**a**^ (95% CI)	n (trials)	Study limitations	Imprecision	Inconsistency	Publication bias	Quality
Main outcome	0.15 (0.05 to 0.25)	1560 (20)	-1^b^	None	None	None	Moderate
Femoral neck BMD	0.09 (− 0.03 to 0.21)	1032 (14)	-1^b^	None	None	-1^c^	Low
Lumbar spine BMD	0.17 (0.04 to 0.30)	874 (11)	-1^b^	None	None	None	Moderate

*BMD* bone mineral density, *n* number of participants

^a^pooled standardised effect size and 95% confidence intervals (positive value favours physical activity interventions)

^b^ > 50% of studies in the meta-analysis had a PEDro score < 6/10

^c^Serious small study effects suggested by visual inspection of funnel plot or sensitivity analysis investigating the impact of removal of small studies on pooled estimate

**Fig. 3:**
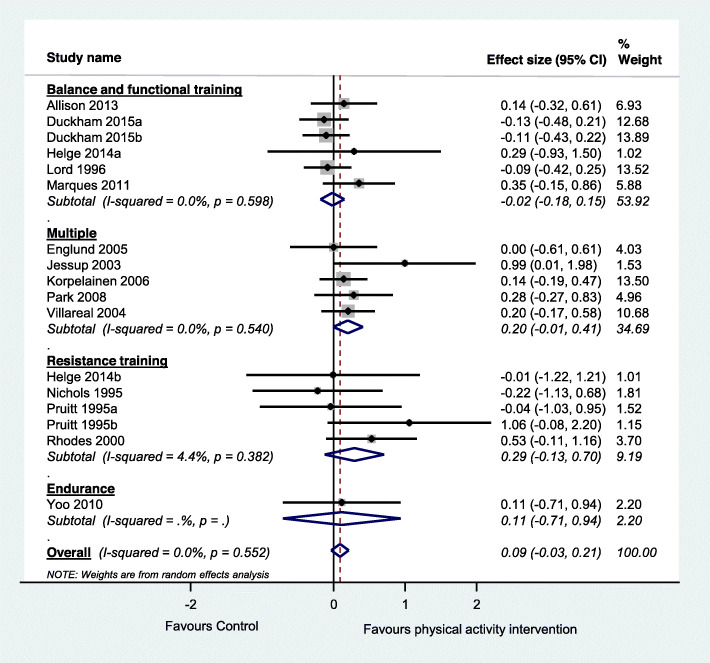
Effect size (95% confidence interval) of physical activity interventions on the femoral neck bone mineral density by pooling data from 14 studies comparing physical activity versus control using random-effects meta-analysis (*n* = 1032)

We also summarised the evidence for the two most commonly reported outcome measures across the included studies. Meta-analysis found a non-significant and small overall effect of physical activity on femoral neck BMD (standardised effect size 0.09, 95% CI − 0.03 to 0.21, 14 trials; Fig. 3). The quality of the evidence was low, downgraded for study limitations and publication bias, suggesting limited confidence in the results (Table 5 and Additional file 5, Supplementary Table B). Overall, these results suggest that physical activity interventions may improve BMD of the femoral neck in older adults.

**Fig. 4:**
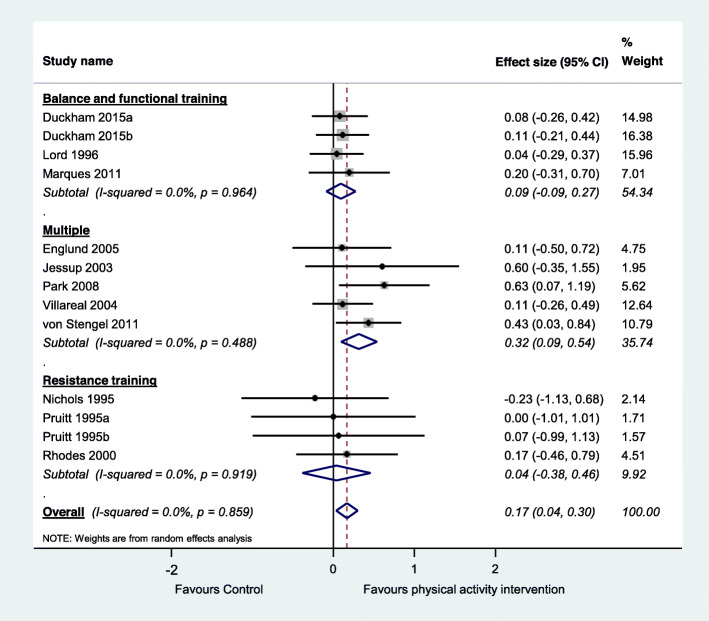
Effect size (95% confidence interval) of physical activity interventions on the lumbar spine bone mineral density by pooling data from 11 studies comparing physical activity versus control using random-effects meta-analysis (*n* = 874)

Lumbar spine BMD was the second mostly commonly reported outcome measures. Meta-analysis found a significant but relatively small overall effect of physical activity on lumbar spine BMD (standardised effect size 0.17, 95% CI 0.04 to 0.30, 11 trials; Fig. 4). The quality of the evidence was moderate, downgraded for study limitations, suggesting that the true effect is likely to be close to the estimated results (Table 5 and Additional file 5, Supplementary Table C). The overall results suggest that physical activity interventions probably improve BMD of the lumbar spine in older adults.

We included 12 observational studies. Since the studies varied in terms of design, statistical approach and measures of physical activity, we did not perform meta-analysis and apply the GRADE approach. Overall, studies showed a positive effect of physical activity on bone health (Table 4).

### Dose response association

As shown in Table 1, programs which had significant impacts were generally of a higher dose. Typical program for which significant intervention impacts were detected in randomised controlled trials were undertaken for 60+ mins, 2–3 times/week for 7+ months [45, 52, 59, 63, 71]. The randomised controlled trials (*n* = 6) investigating different doses of physical activity on bone health did not suggest a clear dose-response relationship (Table 3) but were probably too small (i.e., lacked statistical power) to detect differences between different doses of physical activity. All eight longitudinal studies investigating different doses of total or planned physical activity on bone health found that higher levels of physical activity were associated with better bone health (Table 4).

Meta-regression revealed a non-significant trend for studies with a higher overall intervention dose (i.e., 7800+ total mins) to have greater effects on femoral neck BMD (*p* = 0.144), where high dose interventions (7800+ mins) had a moderate effect with a standardised effect size of 0.26, 95% CI − 0.01 to 0.52 and lower dose interventions (< 7800 mins) had a small effect 0.03, 95% CI − 0.12 to 0.19, although neither sub-group effect was statistically significant. Similar results were found for lumbar spine BMD, where the difference in effects did not reach statistical significance (*p* = 0.373), but higher dose interventions had a moderate effect (standardised effect 0.33, 95% CI − 0.08 to 0.73) whereas lower dose interventions had a small effect (standardised effect 0.14, 95% CI − 0.02 to 0.30), although neither sub-group effect was statistically significant.

### Type and domain of physical activity

Meta-regression was undertaken to investigate whether the inclusion of any particular component in a program was associated with greater overall effects. There was a trend for greater effects of programs that included multiple exercise or resistance types on femoral neck BMD (*p* = 0.059 for the difference in effects) with significant effects for the programs that involved multiple exercise types or resistance exercise (standardised effect 0.24, 95% CI 0.03 to 0.44) but not for programs that did not (standardised effect − 0.02, 95% CI − 0.19 to 0.15). Similarly, there was a trend of greater effects in programs that included multiple exercise and resistance types on lumbar spine BMD (*p* = 0.256 for the difference in effects) with significant effects for the programs that involved multiple exercise types or resistance exercise (standardised effect 0.26, 95% CI 0.04 to 0.48) but not for programs that did not (standardised effect 0.09, 95% CI − 0.11 to 0.30). There was no evidence of differential effects by the inclusion of bone loading exercises or balance exercises.

Meta-analysis revealed that programs including multiple exercise types had a significant impact on bone when the main outcome from each study was pooled (standardised effect size 0.20, 95% CI 0.04 to 0.37, *n* = 8 trials; Fig. 2), as well as on a pooled analysis of lumbar spine BMD (standardised effect size 0.32 95% CI 0.09 to 0.54, *n* = 5 trials; Fig. 4). Meta-analysis findings did not quite reach significance for programs including multiple exercise types for femoral neck BMD (standardised effect size 0.20, 95% CI − 0.01 to 0.41, *n* = 5 trials, Fig. 3).

Meta-analysis revealed that the effects of balance and functional exercises did not reach significance when the main outcome from each study was pooled (Fig. 2), or when femoral neck BMD (Fig. 3) and lumbar spine BMD (Fig. 4) were analysed. Meta-analysis also revealed that the pooled effects of resistance training as a single exercise component was not significant for the overall analysis (Fig. 2), femoral neck BMD (Fig. 3) and lumbar spine BMD outcomes (Fig. 4).

There were 11 studies comparing two or more forms of physical activity interventions (Table 2). The comparisons investigated by the studies included: balance vs resistance [42, 50, 53], balance vs endurance [44], multiple vs resistance [53, 90], 3D vs resistance [69, 75], multiple vs balance [53], endurance vs multiple [67], multiple vs multiple [55] and endurance vs resistance vs multiple [87]. Only one trial found a statistically significant difference when endurance was compared to resistance or to multiple, with results favouring endurance. None of the remaining studies found a statistically significant difference between the groups and there was no clear pattern of superiority of one form of physical activity in relation to the others. Overall the studies investigated a small sample of participants (median = 58; range 23 to 176 participants analysed) and may have lacked statistical power to detect differences between forms of physical activity interventions.

The programs used in the randomised controlled trials that detected such impacts involved weight-bearing exercises that challenged balance and function, plus additional components (such as added resistance and/or endurance training) and were of a relatively high dose (60+ mins, 2+ times per week) and duration (1+ years). For example the study by Bunout [45] involved a 1 h session of chair stands, squats, step-ups in a stair, arm pull-ups, respiratory muscle training with 15-min walking periods before and after these exercises, and was undertaken twice a week, with the intensity graded by a specialised coach using the Borg scale and lasted for 72 weeks. The study by Jessup [52] also involved multiple components, was undertaken three times a week with 60–90 min per session plus 30–45 min of walking and involved resistance training using a weighted vest.

Taken together, these results suggest that interventions involving a combination of multiple exercise types or resistance exercise may improve bone health and prevent osteoporosis in older people.

### Exploration of the impact of study quality

Exploratory meta-regression did not reveal a differential effect of studies that scored less or more than 6 on the PEDro scale (*p* = 0.667).

## Discussion

### Summary of main results

This review includes 59 studies and of these, 20 randomised controlled trials with 1560 participants contributed to the evidence for the comparison of physical activity interventions with control on the main studies outcome. There is moderate quality evidence that physical activity has a significant but small effect on bone health and particularly in lumbar BMD. The level of evidence is lower for femoral neck BMD, where a small and non-significant effect was found. Programs involving higher doses and multiple exercise types or resistance exercise appear to be more effective. Although it is unclear whether an effect of this magnitude is meaningful for clinicians or patients, overall our results suggest that physical activity probably plays a role in the prevention of osteoporosis.

### Interpretation and implications of the findings

The aim of this review was to investigate the effect of physical activity on osteoporosis prevention in older people. However, none of the studies included in this review reported diagnosis of osteoporosis as an outcome measure. The most commonly reported outcome was BMD, which is commonly used to define osteoporosis. According to the WHO criteria, osteoporosis is defined as a BMD that lies 2.5 standard deviations or more below the average value for young healthy women (a T-score of <− 2.5 SD) [100]. Low BMD is one of several risk factors for fractures [101, 102], the main clinical manifestation of osteoporosis. Previous longitudinal studies have indicated the contribution of BMD to fracture, with a one standard deviation decrease in BMD resulting in 2 to 3.5 times greater risk of fracture [103]. A recent individual patient data review including data from 91,779 participants from multiple randomised controlled trials has demonstrated that treatment-related BMD changes are strongly associated with fracture reductions in trials of interventions for osteoporosis, supporting the use of BMD as a surrogate outcome for fracture in randomised controlled trials [104].

Although this review has revealed a small effect of physical activity on bone health, this finding should be interpreted considering the additional benefits of physical activity on other risk factors for fractures in older people, such as falls [105], poor strength [102] and balance [106]. Taken together, these findings suggest that it is likely that physical activity generates clinically meaningful benefits for the prevention of osteoporosis in older people. Clinicians and policy makers should consider these findings when prescribing exercises to older patients without a diagnosis of osteoporosis or making public health decisions.

Although the optimal exercise intervention to prevent osteoporosis has not been defined, our sub-group analysis and meta-regression results suggest that those that included multiple exercises types and resistance exercises had greater effects. These findings are in agreement with a previous review that found that the most effective intervention for spine BMD in postmenopausal women was combination exercise programs (pooled mean difference 3.22; 95% CI 1.80 to 4.64) [17], however this pooled analysis included participants with and without osteoporosis, unlike our review that focused on prevention only.

Our findings also suggested a dose-response relationship with typical programs that showed an impact being undertaken for 60+ min, 2–3 times/week for 7+ months. The studies investigating exercises programs had a median duration of 12 months and it is likely that longer exercise programs would have greater effects on bone health, as suggested by the longitudinal studies. Although the confidence in these findings is not high, these results are in line with guideline recommendations that participants without osteoporosis should engage regularly in physical activity (at least 2–3 times/week) and programs should include a combination of exercises types [107].

Although previous reviews have suggested that bone loading (high impact) exercises and non-weight-bearing high force exercise alone provide benefits to bone health [17, 18, 107], we were not able to confirm these results in this review. Since previous reviews have used different classification systems for physical activity interventions, direct comparisons are not possible. Additionally, in the present review none of the included studies investigated bone loading alone. Other factors that might explain differences in our findings in relation to previous reviews include the fact that previous reviews have investigated younger participants, have pooled together studies investigating the effect of physical activity on prevention (i.e. in participants without osteoporosis) and management (i.e. in participants with osteoporosis at baseline) of osteoporosis.

### Strengths and potential biases in the review process

This review provides a comprehensive overview of the evidence on the role of physical activity on osteoporosis prevention in older people, without limits by gender, body parts, or physical activity type. Additionally, we were able to perform analysis according to physical activity types and to explore the effect of dose on the physical activity effects.

The initial aim of this review was to summarise the evidence of physical activity on prevention of osteoporosis in older people by conducting a review of systematic reviews. However, since no reviews were found we included the relevant studies identified from the reviews. We decided to expand the search for individual studies, since the initial search was targeted at reviews, and it was possible that we had missed important studies, particularly recently-published ones (the most recent included study in the report was published in 2015). We were able to include 19 additional studies with our expanded search. We also updated our search for reviews in PubMed and conducted searches in three additional databases. We found 4 additional studies and although our main results remained unchanged with the addition of these studies, our search was focused on reviews, rather than individual studies, and it is possible that we might have missed relevant studies that were not included in the identified reviews.

We only included studies investigating the effects of physical activity for the prevention of osteoporosis and therefore excluded studies where all participants had been diagnosed with osteoporosis. Most studies did not use the absence of osteoporosis at baseline as an inclusion criterion. Therefore, it is likely that the studies investigated samples of people with mixed bone health status. One review author classified the exercise interventions using the ProFaNE guidelines [30] and a second one checked the classification. We recognise there is some subjectivity in this classification system, particularly for those interventions containing more than one category of exercise.

### Unanswered questions and future research

This review has focused on older people only but it is likely that exposure to physical activity earlier in life plays a key role in bone deposition and thereby, osteoporosis prevention, as indicated by previous studies [108], however this was beyond the scope of this review. We focused on prevention of osteoporosis, and therefore excluded studies where all participants were diagnosed with osteoporosis. Since bone health is a continuum, the inclusion of studies of people with existing osteoporosis would provide additional understanding of the effect of physical activity on osteoporosis but was also beyond the scope of this review. The investigation of the effects of physical activity on fragility fractures was not covered in this review. However, since fragility fracture is the main clinical manifestation of osteoporosis [1], future research should focus on investigating the impact of physical activity on this outcome. Lastly, previous reviews investigating the effects of physical activity programs on osteoporosis have used different classification systems for physical activity. Future studies should focus on using standardised classification systems to facilitate comparison of results across reviews.

The overall quality of included studies varied and overall was low (median PEDro score = 5), and this has been taken into account in the GRADE approach, where all three analysis were downgraded on the basis of study limitations. Although meta-regression did not reveal a differential effect when studies were stratified as high or low quality, future studies should improve the methodological quality of studies, particularly in terms of follow-up rate, allocation concealment and intention to treat analysis, which were the main limitations of studies in this review. Additionally, the trials had a small sample size (median = 50) and relatively short follow-up (median follow-up length = 12 months). Future studies should investigate larger samples and have longer follow-up duration.

## Conclusions

In summary, while the results need to be treated with some caution, the studies included in this review suggest that physical activity is likely to play a role in the prevention of osteoporosis in older people. The level of evidence is higher for lumbar spine BMD (than for femoral neck BMD) and higher dose programs and those involving multiple exercises types or resistance exercise appear to be more effective.

## Supplementary Information

**Additional file 1:** Search strategies and inclusion criteria.

**Additional file 2:** Categories of physical activity programmes (ProFaNE): definitions and application.

**Additional file 3:** Methodological quality of included observational studies.

**Additional file 4:** Methodological quality and reporting of included trials.

**Additional file 5:** Level of evidence according to the GRADE approach (Supplementary tables and figures).

## Data Availability

All data generated or analysed during this study are included in this published article and its supplementary information files.

## Abbreviations

BMC: Bone mineral content
BMD: Bone mineral density
CI: Confidence interval
GDG: Guideline Development Group
GRADE: Grading of Recommendations Assessment, Development and Evaluation
PICO: Population, intervention, comparison, outcome
ProFaNE: Prevention of Falls Network Europe
WHO: World Health Organization
