# Supplementary material for: Evidence on physical activity and osteoporosis prevention for people aged 65+ years: a systematic review to inform the WHO guidelines on physical activity and sedentary behaviour
Source: Int J Behav Nutr Phys Act. 2020 Nov 26;17:150. doi: 10.1186/s12966-020-01040-4 (PMC7690138; doi:10.1186/s12966-020-01040-4)
Supplement: Supplementary file 1 — Additional file 1: Search strategies and inclusion criteria. [file 12966_2020_1040_MOESM1_ESM.docx]

**APPENDIX 1: Search strategies and inclusion criteria**

**A. Search strategy WHO report**

Database: PubMed

Date of Search: 06/11/2019

| **Description** | **Search terms*** |
| --- | --- |
| Limit: language and exclude animal only | (English[lang]) NOT ("Animals"[Mesh] NOT ("Animals"[Mesh] AND "Humans"[Mesh])) |
| Limit: age groups | NOT (("infant"[Mesh] OR "child"[mesh] OR "adolescent"[mh]) NOT (("infant"[Mesh] OR "child"[mesh] OR "adolescent"[mh]) AND "adult"[Mesh])) |
| Limit: date | AND ("2008/01/01"[PDAT] : "3000/12/31"[PDAT]) |
| Publication type | AND (systematic[sb] OR meta-analysis[pt] OR “systematic review”[tiab] OR “systematic literature review”[tiab] OR metaanalysis[tiab] OR "meta analysis"[tiab] OR metanalyses[tiab] OR "meta analyses"[tiab] OR "pooled analysis"[tiab] OR “pooled analyses”[tiab] OR "pooled data"[tiab]) |
| Limit: publication type | NOT (“comment”[Publication Type] OR “editorial”[Publication Type]) |
| Physical activity | AND (("Exercise"[mh] OR "Exercise"[tiab] OR "Physical activity"[tiab] OR "Sedentary behavior"[mh] OR "Lifestyle activities"[tiab] OR "Lifestyle activity"[tiab] OR "Recreational activities"[tiab] OR "Recreational activity"[tiab] OR "Tai ji"[mh] OR "Yoga"[mh] OR "Activities of daily living"[tiab] OR "Activity of daily living"[tiab] OR "Free living activities"[tiab] OR "Free living activity"[tiab] OR "Balance training"[tiab] OR "Qigong"[mh] OR "Functional training"[tiab]) OR (("Aerobic activities"[tiab] OR "Aerobic activity"[tiab] OR "Cardiovascular activities"[tiab] OR "Cardiovascular activity"[tiab] OR "Endurance activities"[tiab] OR "Endurance activity"[tiab] OR "Physical activities"[tiab] OR "Physical conditioning"[tiab] OR "Resistance training"[tiab] OR "strength training"[tiab] OR "Sedentary"[tiab] OR "Tai chi"[tiab] OR "Tai ji"[tiab] OR "Yoga"[tiab] OR "Walk"[tiab] OR "Walking"[tiab] OR "Chi kung"[tiab] OR "Qigong"[tiab] OR "stretching"[tiab]) NOT medline[sb])) |
| Osteoporosis | AND ("Osteoporosis"[mh] OR “Osteoporosis”[tiab] OR “Osteoporoses”[tiab] OR “Post-Traumatic Osteoporosis” [tiab] OR “Senile Osteoporosis” [tiab] OR “Age-Related Bone Loss” [tiab] OR “Age-Related Bone Losses” [tiab] OR “Age-Related Osteoporosis” [tiab] OR “Age Related Osteoporosis” [tiab] OR “Age-Related Osteoporoses” [tiab]) |

*Search strategy created by Guideline Development Group

**B. Expanded search strategy for individual studies**

Database: PubMed

Date of search: 11 March 2020

| **Description** | **Search terms*** |
| --- | --- |
| Limit: language and exclude animal only | (English[lang]) NOT ("Animals"[Mesh] NOT ("Animals"[Mesh] AND "Humans"[Mesh])) |
| Limit: age groups | NOT (("infant"[Mesh] OR "child"[mesh] OR "adolescent"[mh]) NOT (("infant"[Mesh] OR "child"[mesh] OR "adolescent"[mh]) AND "adult"[Mesh])) |
| Limit: date | AND ("2010/01/01"[PDAT]: "3000/12/31"[PDAT]) |
| Publication type | AND (((((((((randomized controlled trial[Publication Type]) OR controlled clinical trial[Publication Type]) OR randomi#ed[Title/Abstract]) OR placebo[Title/Abstract]) OR sham[Title/Abstract]) OR randomly[Title/Abstract]) OR "clinical trials as topic"[MeSH Terms]) OR trial[Title])) OR ((((((((((((("cohort studies"[MeSH Terms]) OR "cohort studies"[Text Word]) OR "cohort study"[Text Word]) OR "longitudinal studies"[MeSH Terms]) OR "longitudinal study"[Text Word]) OR "longitudinal studies"[Text Word]) OR "follow up studies"[MeSH Terms]) OR "follow up study"[Text Word]) OR "follow up studies"[Text Word]) OR "follow up"[Text Word]) OR "prospective studies"[MeSH Terms]) OR "prospective study"[Text Word]) OR "prospective studies"[Text Word]) |
| Limit: publication type | NOT (“comment”[Publication Type] OR “editorial”[Publication Type]) |
| Physical activity | AND (("Exercise"[mh] OR "Exercise"[tiab] OR "Physical activity"[tiab] OR "Sedentary behavior"[mh] OR "Lifestyle activities"[tiab] OR "Lifestyle activity"[tiab] OR "Recreational activities"[tiab] OR "Recreational activity"[tiab] OR "Tai ji"[mh] OR "Yoga"[mh] OR "Activities of daily living"[tiab] OR "Activity of daily living"[tiab] OR "Free living activities"[tiab] OR "Free living activity"[tiab] OR "Balance training"[tiab] OR "Qigong"[mh] OR "Functional training"[tiab]) OR (("Aerobic activities"[tiab] OR "Aerobic activity"[tiab] OR "Cardiovascular activities"[tiab] OR "Cardiovascular activity"[tiab] OR "Endurance activities"[tiab] OR "Endurance activity"[tiab] OR "Physical activities"[tiab] OR "Physical conditioning"[tiab] OR "Resistance training"[tiab] OR "strength training"[tiab] OR "Sedentary"[tiab] OR "Tai chi"[tiab] OR "Tai ji"[tiab] OR "Yoga"[tiab] OR "Walk"[tiab] OR "Walking"[tiab] OR "Chi kung"[tiab] OR "Qigong"[tiab] OR "stretching"[tiab]) NOT medline[sb])) |
| Osteoporosis | AND ("Osteoporosis"[mh] OR “Osteoporosis”[tiab] OR “Osteoporoses”[tiab] OR “Post-Traumatic Osteoporosis” [tiab] OR “Senile Osteoporosis” [tiab] OR “Age-Related Bone Loss” [tiab] OR “Age-Related Bone Losses” [tiab] OR “Age-Related Osteoporosis” [tiab] OR “Age Related Osteoporosis” [tiab] OR “Age-Related Osteoporoses” [tiab]) |

*Search strategy created by Guideline Development Group and adapted by the review authors to target individual studies

**C. Expanded search strategy for systematic reviews**

C.1. Search strategy PubMed

Date of Search: 07/07/2020

| **Description** | **Search terms*** |
| --- | --- |
| Limit: language and exclude animal only | (English[lang]) NOT ("Animals"[Mesh] NOT ("Animals"[Mesh] AND "Humans"[Mesh])) |
| Limit: age groups | NOT (("infant"[Mesh] OR "child"[mesh] OR "adolescent"[mh]) NOT (("infant"[Mesh] OR "child"[mesh] OR "adolescent"[mh]) AND "adult"[Mesh])) |
| Limit: date | AND ("2019/01/01"[PDAT] : "3000/12/31"[PDAT]) |
| Publication type | AND (systematic[sb] OR meta-analysis[pt] OR “systematic review”[tiab] OR “systematic literature review”[tiab] OR metaanalysis[tiab] OR "meta analysis"[tiab] OR metanalyses[tiab] OR "meta analyses"[tiab] OR "pooled analysis"[tiab] OR “pooled analyses”[tiab] OR "pooled data"[tiab]) |
| Limit: publication type | NOT (“comment”[Publication Type] OR “editorial”[Publication Type]) |
| Physical activity | AND (("Exercise"[mh] OR "Exercise"[tiab] OR "Physical activity"[tiab] OR "Sedentary behavior"[mh] OR "Lifestyle activities"[tiab] OR "Lifestyle activity"[tiab] OR "Recreational activities"[tiab] OR "Recreational activity"[tiab] OR "Tai ji"[mh] OR "Yoga"[mh] OR "Activities of daily living"[tiab] OR "Activity of daily living"[tiab] OR "Free living activities"[tiab] OR "Free living activity"[tiab] OR "Balance training"[tiab] OR "Qigong"[mh] OR "Functional training"[tiab]) OR (("Aerobic activities"[tiab] OR "Aerobic activity"[tiab] OR "Cardiovascular activities"[tiab] OR "Cardiovascular activity"[tiab] OR "Endurance activities"[tiab] OR "Endurance activity"[tiab] OR "Physical activities"[tiab] OR "Physical conditioning"[tiab] OR "Resistance training"[tiab] OR "strength training"[tiab] OR "Sedentary"[tiab] OR "Tai chi"[tiab] OR "Tai ji"[tiab] OR "Yoga"[tiab] OR "Walk"[tiab] OR "Walking"[tiab] OR "Chi kung"[tiab] OR "Qigong"[tiab] OR "stretching"[tiab]) NOT medline[sb])) |
| Osteoporosis | AND ("Osteoporosis"[mh] OR “Osteoporosis”[tiab] OR “Osteoporoses”[tiab] OR “Post-Traumatic Osteoporosis” [tiab] OR “Senile Osteoporosis” [tiab] OR “Age-Related Bone Loss” [tiab] OR “Age-Related Bone Losses” [tiab] OR “Age-Related Osteoporosis” [tiab] OR “Age Related Osteoporosis” [tiab] OR “Age-Related Osteoporoses” [tiab]) |

*Search strategy created by Guideline Development Group

C.2. Search strategy Embase (via Ovid)

Date of Search: 07/07/2020

1. exp exercise/
2. exercis*.tw.
3. exp physical activity/
4. exp sedentary lifestyle/
5. exp sport/
6. exp Tai Chi/
7. exp Yoga/
8. exp daily life activity/
9. exp qigong/
10. physical activit*.tw.
11. "Lifestyle activit*".ti,ab.
12. "Recreational activit*".ti,ab.
13. "Activit* of daily living".ti,ab.
14. "Free living activit*".ti,ab.
15. "Balance training".ti,ab.
16. "Functional training".ti,ab.
17. "Aerobic activit*".ti,ab.
18. "Cardiovascular activit*".ti,ab.
19. "Endurance activit*".ti,ab.
20. "Physical activit*".ti,ab.
21. "Physical conditioning".ti,ab.
22. "Resistance training".ti,ab.
23. "strength training".ti,ab.
24. "Sedentary".ti,ab.
25. "Tai chi".ti,ab.
26. "Tai ji".ti,ab.
27. Yoga.ti,ab.
28. walk.ti,ab.
29. walking.ti,ab.
30. "Chi Kung".ti,ab.
31. Qigong.ti,ab.
32. stretching.ti,ab.
33. 1-32 OR
34. exp osteoporosis/
35. osteoporo$.tw.
36. Osteoporos$.ti,ab.
37. "Post Traumatic Osteoporosis".ti,ab.
38. "Senile Osteoporosis".ti,ab.
39. "Age-Related Bone Loss$".ti,ab.
40. "Age-Related Osteoporo$".ti,ab.
41. Bone loss$.tw.
42. exp bone density/
43. 37-45 OR
44. exp "systematic review"/
45. exp meta analysis/
46. meta analys$.ti,ab.
47. "systematic review".ti,ab.
48. "systematic literature review".ti,ab.
49. metaanalys$.ti,ab.
50. "meta analys$".ti,ab.
51. "pooled analys$".ti,ab.
52. "pooled data".ti,ab.
53. 47-54 OR
54. 36 AND 46 AND 55
55. Limit 53 to (human and English language and yr= “2008-Currrent”)
56. Limit 54 to (adult <18 to 64 years> or aged <65+years>)

Observation: search strategy created by the authors based on the search created by the Guideline Development Group for PubMed.

C.3. Search strategy CINAHL

Date of Search: 07/07/2020

1. (MH "Exercise+")
2. TI exercise OR AB exercise
3. TI "physical activity" OR AB "physical activity"
4. (MH "Life Style, Sedentary+")
5. TI "Lifestyle activities" OR AB "Lifestyle activities" OR TI "Lifestyle activity" OR AB "Lifestyle activity"
6. TI "Recreational activities" OR AB "Recreational activities" OR TI "Recreational activity" OR AB "Recreational activity"
7. (MH "Tai Chi")
8. (MH "Yoga+")
9. (MH "Activities of Daily Living+")
10. TI "Activities of daily living" OR AB "Activities of daily living"
11. TI "Free living activity" OR AB "Free living activity"
12. TI "Balance training" OR AB "Balance training"
13. (MH "Qigong")
14. TI "Functional training" OR AB "Functional training"
15. TI "Aerobic activities" OR AB "Aerobic activities" OR TI "Aerobic activity" OR AB "Aerobic activity"
16. TI "Cardiovascular activities" OR AB "Cardiovascular activities" OR TI "Cardiovascular activity" OR AB "Cardiovascular activity"
17. TI "Endurance activities" OR AB "Endurance activities" OR TI "Endurance activity" OR AB "Endurance activity"
18. TI "Physical activities" OR AB "Physical activities"
19. TI "Physical conditioning" OR AB "Physical conditioning"
20. TI "Resistance training" OR AB "Resistance training"
21. TI "strength training" OR AB "strength training"
22. TI "Sedentary" OR AB "Sedentary"
23. TI "Tai chi" OR AB "Tai chi" OR TI "Tai ji" OR AB "Tai ji"
24. TI Yoga OR AB Yoga
25. TI Walk OR AB Walk OR TI walking OR AB walking
26. TI "Chi kung" OR AB "Chi kung" OR TI "Qigong" OR AB "Qigong"
27. TI stretching OR AB stretching
28. 1-27 OR
29. (MH "Osteoporosis+")
30. TI Osteoporosis OR AB Osteoporosis
31. TI Osteoporoses OR AB Osteoporoses
32. TI “Senile Osteoporosis” OR AB “Senile Osteoporosis” ‘
33. TI “Age-Related Bone Loss” OR AB “Age-Related Bone Loss”
34. TI “Age-Related Osteoporosis” OR AB “Age-Related Osteoporosis”
35. TI “Age Related Osteoporosis” OR AB “Age Related Osteoporosis”
36. 29-35 OR
37. (MH "Systematic Review")
38. TI systematic review OR AB systematic review
39. TI systematic literature review OR AB systematic literature review
40. TI metaanalysis OR AB metaanalysis OR TI meta analysis OR AB meta analysis
41. TI metaanalyses OR AB metaanalyses OR TI meta analyses OR AB meta analyses
42. TI meta-analysis OR AB meta-analysis OR TI meta-analyses OR AB meta-analyses
43. TI "pooled analysis" OR AB "pooled analysis" OR TI "pooled analyses" OR AB "pooled analyses"
44. TI "pooled data" OR AB "pooled data"
45. 37-44 OR
46. 28 AND 36 AND 45
47. Limit to English AND Limit to Published Date: 20080101-20201231

Observation: search strategy created by the authors based on the search created by the Guideline Development Group for PubMed.

## C.4. Search strategy **SPORTDiscus (via EBSCO)**

Date of Search: 07/07/2020

1. Exercise
2. TI exercise OR AB exercise
3. TI "physical activity" OR AB "physical activity"
4. sedentary behavior
5. TI "Lifestyle activities" OR AB "Lifestyle activities" OR TI "Lifestyle activity" OR AB "Lifestyle activity"
6. TI "Recreational activities" OR AB "Recreational activities" OR TI "Recreational activity" OR AB "Recreational activity"
7. Tai ji OR Tai Chi
8. Yoga
9. TI "Activities of daily living" OR AB "Activities of daily living" OR TI "Activity of daily living" OR AB "Activity of daily living"
10. TI "Free living activities" OR AB "Free living activities" OR TI "Free living activity" OR AB "Free living activity"
11. TI "Balance training" OR AB "Balance training"
12. Qigong
13. TI "Functional training" OR AB "Functional training"
14. TI "Aerobic activities" OR AB "Aerobic activities" OR TI "Aerobic activity" OR AB "Aerobic activity"
15. TI "Cardiovascular activities" OR AB "Cardiovascular activities" OR TI "Cardiovascular activity" OR AB "Cardiovascular activity"
16. TI "Endurance activities" OR AB "Endurance activities" OR TI "Endurance activity" OR AB "Endurance activity"
17. TI "Physical activities" OR AB "Physical activities"
18. TI "Physical conditioning" OR AB "Physical conditioning"
19. TI "Resistance training" OR AB "Resistance training"
20. TI "strength training" OR AB "strength training"
21. TI sedentary OR AB sedentary
22. TI "Tai chi" OR AB "Tai chi" OR TI "Tai ji" OR AB "Tai ji"
23. TI Yoga OR AB Yoga
24. TI walk OR AB walk OR TI walking OR AB walking
25. TI "Chi kung" OR AB "Chi kung" OR TI Qigong OR AB Qigong
26. TI stretching OR AB stretching
27. 1-26 OR
28. osteoporosis
29. TI osteoporosis OR AB osteoporosis OR TI osteoporoses OR AB osteoporoses
30. TI “Senile Osteoporosis” OR AB “Senile Osteoporosis”
31. TI “Age-Related Bone Loss” OR AB “Age-Related Bone Loss” OR TI “Age-Related Bone Losses” OR AB “Age-Related Bone Losses”
32. TI “Age-Related Osteoporosis” OR AB “Age-Related Osteoporosis” OR TI “Age-Related Osteoporoses” OR AB “Age-Related Osteoporoses”
33. TI “Age Related Osteoporosis” OR AB “Age Related Osteoporosis” OR TI “Age Related Osteoporoses” OR AB “Age Related Osteoporoses”
34. 28-34 OR
35. systematic review
36. meta-analysis
37. TI systematic review OR AB systematic review
38. TI “systematic literature review” OR AB “systematic literature review”
39. TI metaanalysis OR AB metaanalysis OR TI metanalyses OR AB metanalyses
40. TI "meta analysis" OR AB "meta analysis" OR TI "meta analyses" OR AB "meta analyses"
41. TI "pooled analysis" OR AB "pooled analysis" OR TI "pooled analyses" OR AB "pooled analyses"
42. TI "pooled data" OR AB "pooled data"
43. 36-42 OR
44. S27 AND S34 and S43
45. Limit to English AND Limit Publication date: 20080101-20201231

Observation: search strategy created by the authors based on the search created by the Guideline Development Group for PubMed.

**D.** **Eligibility criteria**

| **Category** | **Inclusion criteria** | **Exclusion criteria** |
| --- | --- | --- |
| **Publication language** | - Studies published with full text in English |  |
| **Publication date** | - Reviews published 2008 to present |  |
| **Publication status** | - Studies published in peer-reviewed journals | - Grey literature, including unpublished data, abstracts, conference proceedings |
| **Study design** | - Systematic review - Meta-analyses | - Narrative reviews - Commentaries - Editorials - Systematic review protocols |
| **Characteristics of individual studies** | | |
| **Study design of studies included in the reviews** | - Randomised controlled trials - Non-randomised controlled trials - Prospective cohort studies - Retrospective cohort studies - Systematic reviews - Meta-analyses | - Cross-sectional studies - Before-and-after studies |
| **Study subjects** | - Human subjects - Adults over 64 years of age - Trials that included younger participants were included if the mean age minus one standard deviation was more than 64 years - No restriction will be applied to participants’ health status or setting | - Participants with osteoporosis at baseline. |
| **Exposure / Intervention** | - All types and intensities of physical activity - Studies where participants received multiple interventions may be included if the only difference between the groups was the physical activity intervention | - Studies that only used physical activity as a confounding variable - Studies of multimodal interventions where physical activity is not the main component, or that do not present data on physical activity alone |
| **Comparison** | No physical activity or lesser volume, duration, frequency, or intensity of physical activity. |  |
| **Outcome** | **Include studies in which the outcome is**   - Osteoporosis, including (but not limited to):   - bone mineral density from any location (e.g., neck of femur, spine)   - bone mineral content   - Calcium bone index   - Cortical bone density   - Bone quality index | - Fracture |
