# Supplementary material for: Evidence on physical activity and osteoporosis prevention for people aged 65+ years: a systematic review to inform the WHO guidelines on physical activity and sedentary behaviour
Source: Int J Behav Nutr Phys Act. 2020 Nov 26;17:150. doi: 10.1186/s12966-020-01040-4 (PMC7690138; doi:10.1186/s12966-020-01040-4)
Supplement: Supplementary file 3 — Additional file 3: Methodological quality of included observational studies. [file 12966_2020_1040_MOESM3_ESM.docx]

**Appendix 3. Methodological quality of included observational studies**

| **Domain and Prompting items for Consideration*** | **Ratings** |
| --- | --- |
| **Study Participation**  a. Adequate participation in the study by eligible persons  b. Description of the source population or population of interest  c. Description of the baseline study sample  d. Adequate description of the sampling frame and recruitment  e. Adequate description of the period and place of recruitment  f. Adequate description of inclusion and exclusion criteria | **High bias:** > 2 items poorly rated  **Moderate bias**: 1 or 2 items poorly rated  **Low bias**: no item poorly rated |
| **Study Attrition***  a. Adequate response rate for study participants  b. Description of attempts to collect information on participants who dropped out  c. Reasons for loss to follow-up are provided  d. Adequate description of participants lost to follow-up  e. There are no important differences between participants who completed the study and those who did not | **High bias:** > 2 items poorly rated  **Moderate bias**: 1 or 2 items poorly rated  **Low bias**: no item poorly rated |
| **Exposure Measurement:** | |
| a. A clear definition or description of physical activity is provided  b. Method of physical activity measurement is adequately valid and reliable  c. The method and setting of measurement of physical activity is the same for all study participants  d. Adequate proportion of the study sample has complete data for physical activity  e. Appropriate methods of imputation are used | **High bias**: > 2 items poorly rated  **Moderate bias**: Self-reported tools used to assess physical activity and/or 1 or 2 items poorly rated.  **Low bias**: Objective measure used to assess physical activity and no item poorly rated. |
| **Outcome Measurement:** | |
| a. A clear definition of the outcome is provided  b. Method of outcome measurement used is adequately valid and reliable  c. The method and setting of outcome measurement is the same for all study participants | **High bias**: > 1 items poorly reported  **Moderate bias**: 1 item poorly reported  **Low bias**: no item poorly reported |
| **Study Confounding:** | |
| a. Important confounders are measured  b. Measurement of important confounders is adequately valid and reliable  c. The method and setting of confounding measurement are the same for all study participants  d. Appropriate methods are used if imputation is used for missing confounder data  e. Important potential confounders are accounted for in the study design  f. Important potential confounders are accounted for in the analysis | **High bias:** > 2 items poorly rated  **Moderate bias**: 1 or 2 items poorly rated  **Low bias**: no item poorly rated |
| **Statistical Analysis and Reporting**  a. Sufficient presentation of data to assess the adequacy of the analysis  b. Strategy for model building is appropriate and is based on a conceptual framework or model  c. The selected statistical model is adequate for the design of the study  d. There is no selective reporting of results | **High bias:** > 2 items poorly rated  **Moderate bias**: 1 or 2 items poorly rated  **Low bias**: no item poorly rated |
| **Overall Rating**  **Low Risk of Bias**: Low risk of bias on at least four of the six domains including study confounding. | |

*Methodological quality was assessed using a modified version of the Quality in Prognosis Studies (QUIPS) tool
