# Supplementary material for: Evidence on physical activity and osteoporosis prevention for people aged 65+ years: a systematic review to inform the WHO guidelines on physical activity and sedentary behaviour
Source: Int J Behav Nutr Phys Act. 2020 Nov 26;17:150. doi: 10.1186/s12966-020-01040-4 (PMC7690138; doi:10.1186/s12966-020-01040-4)
Supplement: Supplementary file 4 — Additional file 4: Methodological quality and reporting of included trials. [file 12966_2020_1040_MOESM4_ESM.docx]

# **Appendix 4, Table 1. Methodological quality and reporting of included trials**

| **Study** | **PEDro Scale Items^a^** | | | | | | | | | | | **PEDro Score**  **(0 to 10)** |
| --- | --- | --- | --- | --- | --- | --- | --- | --- | --- | --- | --- | --- |
|  | **1^b^** | **2** | **3** | **4** | **5** | **6** | **7** | **8** | **9** | **10** | **11** |  |
| Allison et al. (2013) | Y | Y | N | Y | N | N | Y | N | N | Y | Y | 5 |
| *†Armamento-Villareal et al. (2020) | Y | Y | N | Y | N | N | Y | Y | Y | Y | Y | 7 |
| †Armamento-Villareal et al. (2012) | Y | Y | N | Y | N | N | Y | Y | Y | Y | Y | 7 |
| Ashe et al. (2013) | Y | Y | Y | Y | N | N | Y | Y | N | N | Y | 6 |
| †Bemben et al. (2011) | Y | Y | N | N | N | N | N | Y | N | Y | Y | 4 |
| Binder et al. (2004) | Y | Y | N | Y | N | N | Y | Y | Y | Y | Y | 7 |
| Blumenthal et al. (1991) | N | Y | N | N | N | N | Y | Y | Y | Y | Y | 6 |
| Bunout et al. (2001) | Y | Y | N | Y | N | N | N | N | N | Y | Y | 4 |
| †Chan et al. (2018) | N | Y | Y | Y | N | N | Y | Y | N | Y | Y | 7 |
| de Jong et al. (2000) | N | Y | Y | Y | N | N | N | N | N | Y | Y | 5 |
| Duckham et al. (2015) | Y | Y | N | Y | N | N | Y | Y | N | Y | Y | 6 |
| Englund et al. (2005) | Y | Y | N | Y | N | N | N | Y | N | Y | Y | 5 |
| Helge et al. (2014) | Y | Y | N | Y | N | N | N | Y | N | Y | Y | 5 |
| Jessup et al. (2003) | Y | Y | N | N | N | N | Y | Y | N | Y | Y | 5 |
| ^Karinkanta et al. (2008) | Y | Y | N | Y | N | N | N | N | Y | Y | Y | 5 |
| Karinkanta et al. (2007) | Y | Y | Y | Y | N | N | N | Y | Y | Y | Y | 7 |
| Kemmler et al. (2010) | Y | Y | N | Y | N | N | Y | N | Y | Y | Y | 6 |
| *†Kemmler et al. (2016) | Y | N | N | Y | N | N | Y | N | N | Y | Y | 4 |
| *†Kemmler et al. (2012) | Y | N | N | Y | N | N | Y | N | N | Y | Y | 4 |
| †Kim et al. (2018) | Y | Y | Y | Y | N | N | Y | N | N | Y | Y | 6 |
| *Kohrt et al. (1997) | N | N | N | Y | N | N | N | N | N | Y | Y | 3 |
| †Korpelainen et al. (2010) | Y | Y | Y | Y | N | N | Y | N | Y | Y | Y | 7 |
| †Korpelainen et al. (2006) | Y | Y | N | Y | N | N | Y | N | Y | Y | Y | 6 |
| *Kwon et al. (2008) | Y | N | N | Y | N | N | N | N | N | Y | Y | 3 |
| Lau et al. (1992) | Y | Y | N | Y | N | N | N | N | N | Y | Y | 4 |
| Lord et al. (1996) | Y | Y | N | Y | N | N | N | N | N | Y | Y | 4 |
| Marques et al. (2011) | Y | Y | Y | Y | N | N | N | N | N | Y | Y | 5 |
| McCartney et al. (1995) | N | Y | N | Y | N | N | N | N | N | Y | N | 3 |
| McMurdo et al. (1997) | Y | Y | N | Y | N | N | N | N | N | Y | Y | 4 |
| ^Nichols et al. (1995) | N | Y | N | Y | N | N | N | N | N | Y | Y | 4 |
| Paillard et al. (2004) | N | Y | N | Y | N | N | N | Y | N | Y | Y | 5 |
| Park et al. (2008) | Y | Y | N | Y | N | N | N | Y | N | Y | Y | 5 |
| Pruitt et al. (1995) | Y | Y | N | Y | N | N | N | N | N | Y | Y | 4 |
| Rhodes et al. (2000) | Y | Y | N | Y | N | N | N | Y | N | Y | Y | 5 |
| *Rikli et al. (1990) | N | N | N | N | N | N | N | N | N | Y | N | 1 |
| Sakai et al. (2010) | N | Y | N | Y | N | N | N | N | N | Y | Y | 4 |
| Shen et al. (2007) | Y | Y | N | Y | N | N | Y | Y | N | Y | Y | 6 |
| *Smith et al. (1981) | Y | N | N | N | N | N | N | N | N | Y | Y | 2 |
| ^Snow et al. (2000) | N | N | N | Y | N | N | N | N | N | Y | Y | 3 |
| Taaffe et al. (1999) | Y | Y | N | Y | N | N | N | Y | N | Y | Y | 5 |
| ^Taaffe et al. (1996) | N | Y | N | Y | N | N | N | N | N | Y | Y | 4 |
| *Villareal et al. (2003) | Y | N | N | N | N | N | N | Y | Y | Y | Y | 4 |
| *Villareal et al. (2004) | Y | Y | N | Y | N | N | N | N | N | Y | Y | 4 |
| von Stengel et al. (2011) | Y | Y | Y | Y | N | N | N | Y | Y | Y | Y | 7 |
| †Winters-Stone et al. (2014) | Y | Y | N | Y | N | N | Y | Y | Y | Y | Y | 7 |
| Woo et al. (2007) | Y | Y | N | Y | N | N | Y | Y | N | Y | Y | 6 |
| Yoo et al. (2010) | Y | Y | N | Y | N | N | N | N | N | Y | Y | 4 |
| Y = yes, N = no.  ^a^1 = Eligibility criteria and source of participants, 2 = random allocation, 3 = concealed allocation, 4 = baseline comparability, 5 = blinded participants, 6 = blinded therapists, 7 = blinded assessors, 8 = adequate follow-up, 9 = intention-to-treat analysis, 10 = between-group comparisons, 11 = point estimates and variability.  ^b^Item 1 does not contribute to the total score.  *Indicate studies where reviewers performed the rating as PEDro scores were not available on PEDro database.  †indicates studies that were found in the updated search  ^ indicates studies that were found in the updated search for systematic reviews conducted in July 2020 in PubMed, Embase, CINAHL, SPORTDiscus  Observational studies were not included in this table | | | | | | | | | | | | |

**Appendix 4, Table 2. Methodological quality and reporting of observational studies**

|  | Study participation | Study attrition | Exposure measurement | Outcomes measurement | Confounding | Analysis and reporting | Overall Risk of Bias |
| --- | --- | --- | --- | --- | --- | --- | --- |
| †Bleicher et al., 2013 | Low | Moderate | Moderate | Low | Low | Low | Low |
| †Foley et al., 2010 | Moderate | Moderate | Low | Low | Moderate | Low | High |
| Greendale et al., 1995 | Low | Low | Moderate | Low | Low | Moderate | Low |
| †Gudmundsdottir et al., 2010 | Low | High | High | Low | Moderate | Low | High |
| Huddleston et al., 1980 | High | High | Moderate | Low | Moderate | Moderate | High |
| †Kemmler et al., 2016 | Moderate | Moderate | Moderate | Low | Moderate | Low | High |
| †Muir et al., 2013 | Low | Low | Moderate | Low | Low | Moderate | Low |
| †Nakamura et al., 2012 | Moderate | Moderate | Moderate | Low | Moderate | Moderate | High |
| Rikkonen et al., 2010 | Low | Moderate | Moderate | Low | Low | Low | Low |
| †Rodriguez-Gomez et al., 2019 | Moderate | Moderate | Low | Low | Moderate | Low | High |
| †Shephard et al., 2017 | Moderate | Moderate | Low | Low | Low | Low | Low |
| †Svejme et al., 2014 | Low | Low | Moderate | Low | Moderate | Low | Low |
| †indicates studies that were found in the updated search | | | | | | | |
