# Supplementary material for: Evidence on physical activity and osteoporosis prevention for people aged 65+ years: a systematic review to inform the WHO guidelines on physical activity and sedentary behaviour
Source: Int J Behav Nutr Phys Act. 2020 Nov 26;17:150. doi: 10.1186/s12966-020-01040-4 (PMC7690138; doi:10.1186/s12966-020-01040-4)
Supplement: Supplementary file 5 — Additional file 5: Level of evidence according to the GRADE approach (Supplementary tables and figures). [file 12966_2020_1040_MOESM5_ESM.docx]

# **APPENDIX 5. Level of evidence according to the GRADE approach (Supplementary tables and figures)**

# **Supplementary Table A. Physical activity interventions vs control on the main outcome of the included studies**

The GRADE approach was applied to the pooled analysis of 20 trials investigating the effects of physical activity compared to a control intervention on the main outcome of the study. Pooled standardised effect size 0.15, 95% CI 0.05 to 0.25.

| **Overall result:** Physical activity interventions probably improve bone health and prevent osteoporosis in older adults. | |
| --- | --- |
| **Level of evidence:** Moderate certainty  We are moderately confident in the effect estimate. The true effect is likely to be close to the estimate of the effect, but there is a possibility that it is substantially different. Further research is likely to have an important impact on our confidence in the estimate of effect and may change the estimate. | |
| **Study limitations** | We downgraded the evidence by one level as 15/20 (75%) of studies in the meta-analysis had a PEDro score <6/10. |
| **Imprecision** | We did not downgrade the evidence due to imprecision as the meta-analysis had 1,560 participants analysed. |
| **Inconsistency of results** | We did not downgrade the evidence due to heterogeneity of included studies as the heterogeneity between trials was small (I^2^=0). Additionally, most of the comparisons in the meta-analysis were in the same direction (23/28, 82%). |
| **Indirectness of evidence** | Since we only included similar studies in terms of population, intervention, comparator and outcome, we did not downgrade the evidence based on this criterion. |
| **Publication bias** | We did not downgrade the evidence for publication bias since examination of funnel plot did not suggest serious small study effects (Appendix 2, Supplementary Figure A). Additional sensitivity analysis where small studies (n<50) were excluded from the meta-analysis showed that the effect estimate is reasonably stable in the absence of small studies (pooled standardised effect size 0.12, 95% CI: 0.01 to 0.23). |

Shading indicates the items that were downgraded.

**Supplementary Table B. Physical activity interventions vs control on femoral neck bone mineral density (BMD)**

The GRADE approach was applied to the pooled analysis of 14 trials investigating the effects of physical activity compared to a control intervention on femoral neck bone mineral density (BMD). Pooled standardised effect size 0.09, 95% CI -0.03 to 0.21.

| **Overall result:** Physical activity interventions may improve bone mineral density of the femoral neck in older adults. | |
| --- | --- |
| **Level of evidence:** **Low certainty**  Our confidence in the effect estimate is limited. The true effect may be substantially different from the estimate of the effect. Further research is very likely to have an important impact on our confidence in the estimate of effect and is likely to change the estimate. | |
| **Study limitations** | We downgraded the evidence by one level as 12/14 studies (86%) included in the meta-analysis had a PEDro score <6/10. |
| **Imprecision** | We did not downgrade the evidence due to imprecision as there were 1,032 participants included in the meta-analysis. |
| **Inconsistency of results** | We did not downgrade the evidence due to heterogeneity of included studies as the heterogeneity between trials was small (I^2^=0). |
| **Indirectness of evidence** | Since we only included similar studies in terms of population, intervention, comparator and outcome, we did not downgrade the evidence based on this criterion. |
| **Publication bias** | We downgraded the evidence for publication bias since examination of funnel plot suggested small study effects, although the effect estimate was not markedly changed in the additional sensitivity analysis where small studies (n<50) were excluded from the meta-analysis (pooled standardised effect size = 0.05, 95% CI: -0.09 to 0.19). |

Shading indicates the items that were downgraded.

# **Supplementary Table C. Level of evidence according to the GRADE approach: Physical activity interventions vs control on lumbar spine bone mineral density (BMD)**

The GRADE approach was applied to the pooled analysis of 11 trials investigating the effects of physical activity compared to a control intervention on lumbar spine bone mineral density (BMD). Pooled standardised effect size 0.17, 95% CI 0.04 to 0.30.

| **Overall result:** Physical activity interventions probably improve bone mineral density of the lumbar spine in older adults. | |
| --- | --- |
| **Level of evidence:** Moderate certainty  We are moderately confident in the effect estimate. The true effect is likely to be close to the estimate of the effect, but there is a possibility that it is substantially different. Further research is likely to have an important impact on our confidence in the estimate of effect and may change the estimate. | |
| **Study limitations** | We downgraded the evidence by one level as 9/11 studies (82%) included in the meta-analysis had a PEDro score <6/10. |
| **Imprecision** | We did not downgrade the evidence due to imprecision as there were 874 participants included in the meta-analysis. |
| **Inconsistency of results** | We did not downgrade the evidence due to heterogeneity of included studies as the heterogeneity between trials was small (I^2^=0). Additionally, all 12/13 (92%) comparisons in the meta-analysis were in the same direction. |
| **Indirectness of evidence** | Since we only included similar studies in terms of population, intervention, comparator and outcome, we did not downgrade the evidence based on this criterion. |
| **Publication bias** | We did not downgrade the evidence for publication bias since examination of funnel plot did not suggest serious small study effects (Appendix 2, Supplementary Figure C). Additional sensitivity analysis where small studies (n<50) were excluded from the meta-analysis showed that the effect estimate is reasonably stable in the absence of small studies (pooled standardised effect size 0.18, 95% CI: 0.03 to 0.32). |

**Supplementary Figures A-C**

**Supplementary Figure A.** Funnel plot of standard error by Hedge’s g for the comparison of exercise vs control on the main outcome of the studies.

**Supplementary Figure B.** Funnel plot of standard error by Hedge’s g for the comparison of exercise vs control on femoral neck bone mineral density.

**Supplementary Figure C.** Funnel plot of standard error by Hedge’s g for the comparison of exercise vs control on lumbar spine bone mineral density.
